# Vocal convergence and social proximity shape the calls of the most basal Passeriformes, New Zealand Wrens

**DOI:** 10.1038/s42003-024-06253-y

**Published:** 2024-05-15

**Authors:** Ines G. Moran, Yen Yi Loo, Stilianos Louca, Nick B. A. Young, Annabel Whibley, Sarah J. Withers, Priscila M. Salloum, Michelle L. Hall, Margaret C. Stanley, Kristal E. Cain

**Affiliations:** 1 School of Biological Sciences, University of Auckland, Auckland, 1142 Aotearoa New Zealand; 2 Centre for Biodiversity and Biosecurity, University of Auckland, Auckland, 1142 Aotearoa New Zealand; 3 Department of Biology, University of Oregon, Eugene, 97403-1210 OR USA; 4 Centre for eResearch, University of Auckland, Auckland, 1142 Aotearoa New Zealand; 5 Department of Zoology, University of Otago, Dunedin, 9016 Aotearoa New Zealand; 6 School of BioSciences, University of Melbourne, Melbourne, VIC 3010 Australia; 7 Bush Heritage Australia, Melbourne, VIC 3000 Australia; 8 School of Biological Sciences, University of Western Australia, Perth, WA 6009 Australia

**Keywords:** Animal behaviour, Quantitative trait

## Abstract

Despite extensive research on avian vocal learning, we still lack a general understanding of how and when this ability evolved in birds. As the closest living relatives of the earliest Passeriformes, the New Zealand wrens (Acanthisitti) hold a key phylogenetic position for furthering our understanding of the evolution of vocal learning because they share a common ancestor with two vocal learners: oscines and parrots. However, the vocal learning abilities of New Zealand wrens remain unexplored. Here, we test for the presence of prerequisite behaviors for vocal learning in one of the two extant species of New Zealand wrens, the rifleman (*Acanthisitta chloris*). We detect the presence of unique individual vocal signatures and show how these signatures are shaped by social proximity, as demonstrated by group vocal signatures and strong acoustic similarities among distantly related individuals in close social proximity. Further, we reveal that rifleman calls share similar phenotypic variance ratios to those previously reported in the learned vocalizations of the zebra finch, *Taeniopygia guttata*. Together these findings provide strong evidence that riflemen vocally converge, and though the mechanism still remains to be determined, they may also suggest that this vocal convergence is the result of rudimentary vocal learning abilities.

## Abstract (web summary)

A study suggests that rifleman – one of the two extant species of New Zealand wrens, the most basal Passeriformes in birds – has predispositions for vocal production learning, including group vocal signatures, vocal convergence, and phenotypic variance call ratios similar to vocal learners.

## Introduction

Most vocal animals communicate with innate vocalizations, but a few taxa are capable of vocal production learning – a behavior that provides animals with the learning ability to copy, match, or imitate sound^1^. Species that vocally learn include a wide range of distantly related taxa such as cetaceans^2^, pinnipeds^3^, elephants^4^, bats^5^, humans, hummingbirds^6^, parrots^7^, songbirds^8^, and although more research is needed, it appears they also include a few suboscines (e.g. bellbirds, *Procnias* spp^9^.), African naked mole-rats (*Heterocephalus glaber*)^10^, musk ducks (*Biziura lobata*)^11^ and black-headed gulls (*Larus ridibundus*), among others^12^. This paraphyly of vocal learners has led to many hypotheses about the evolution of vocal learning, along with a relatively new hypothesis, which suggests that vocal production learning exists along a continuum consisting of modules^1,13^ – as opposed to a binary dichotomy between vocal learners and vocal non-learners (absence *vs*. presence). In this hypothesis, vocal production learning is made up of distinct, yet connected behavioral modules (e.g. vocal convergence, vocal matching, mimicry, and song sharing) – resulting in varying levels of vocal learning complexity (i.e., absent, limited/rudimentary, advanced)^1,14–17^.

**Fig. 1:**
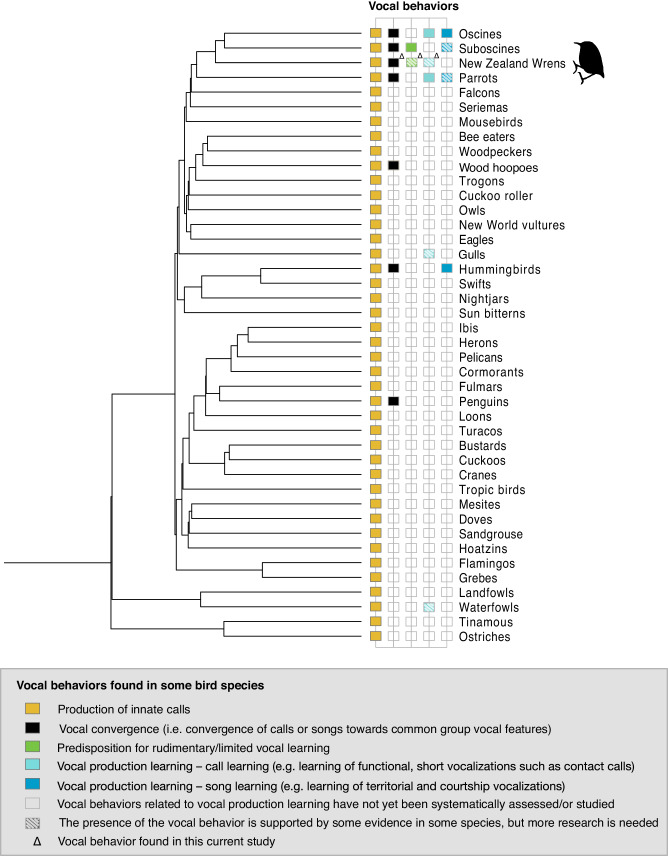
Schematic phylogenetic tree with associated vocal behaviors of New Zealand wrens and other birds. Avian vocal behaviors are categorized based on neurobiological and/or behavioral studies or anecdotes (see below) and are represented with colored rectangles. Behaviors, such as vocal production learning for calls and songs, are separated due to their distinct neurological and functional basis, which may have evolved separately^131,132^. Diagonally barred rectangles indicate that, although there is some evidence for vocal production learning in this taxon, further testing is needed. When no data is available on the presence or absence of vocal production learning in a clade, vocal behaviors are represented with empty rectangles. Triangles highlight novel and previously unknown behaviors found in this current study (i.e. in the rifleman). Innate vocalizations (labeled in yellow) are present in all bird clades suggesting that they were likely present in the common ancestor to all birds. Seven bird clades vocally converge toward common group vocal signatures (labeled in black) and include oscines^41,133,134^, suboscines^101^, New Zealand wrens (based on current study), parrots^44,76,135^, wood-hoopoes^76^, hummingbirds^82^ and penguins^92^. Two bird clades have possible rudimentary/limited vocal learning predispositions (labeled in green), the suboscines – based on neurological evidence^14,20^ and New Zealand Wrens – based on the quantitative genetic model comparisons from this study. Call learning (labeled in light blue) has been demonstrated in oscines^42,51–53^, parrots^56^ and although more research is needed, it is also present in one species of gulls^12,136^ and ducks^11,12^ and may be present in New Zealand Wrens (this study). While song learning (labeled in cyan blue) has clearly been demonstrated in oscines^8,137^ and hummingbirds^6,138^, other forms of song learning have also been found in suboscines^9^ and parrots^139–141^. This simplified subtree was derived from Jetz et al.^142^.

Birds are an excellent group to explore this hypothesis due to their diverse vocal production learning abilities. While advanced vocal learning is well established in parrots (Psittaciformes)^7^, hummingbirds (Trochiliformes)^6^, and oscine songbirds (Passeriformes)^8^, the picture is less clear for suboscines (Passeriformes) and the New Zealand wrens (Passeriformes and sister sub-order to oscines and suboscines, Fig. 1). Suboscines have traditionally been classified as vocal non-learners^18,19^, but some species have been reported as vocal learners^9^, or as limited learners with a rudimentary neural circuitry related to vocal learning in oscine songbirds^14,20^. As for New Zealand wrens, their vocal learning abilities have never been directly tested, and have been assumed to be nonexistent based on their simple syrinx morphology (i.e., lacking the intrinsic muscles present in vocal learners^21,22^), and their basic and short call structure (i.e. lacking complex and broadcast songs^23–25^). However, recent revisions of the avian phylogeny show that the New Zealand wrens share a close common ancestor with vocal learning parrots and oscines, and with suboscines^26–30^ (Fig. 1). According to the continuum/module hypotheses^1,15^, this opens the possibility for New Zealand wrens to have rudimentary learning abilities. Investigating New Zealand wrens’ vocal behaviour and plasticity, and determining where this species fits into the rudimentary/vocal learning continuum hypotheses is hence key to resolve gaps in the evolution of vocal production learning in Passeriformes.

In wild and at-risk animal populations, such as New Zealand wrens, well-established approaches that enable the detection of vocal production learning abilities (e.g., cross-fostering, social isolation, deafening)^31–33^ are often unfeasible. Alternative innovative methods are needed, such as the detection of behavioral modules and behavioral predispositions unique to vocal production learning. An example of such a behavioral module is vocal convergence – a form of vocal modification controlled and maintained over time among socially close conspecifics. To achieve vocal convergence, individuals change their unique vocal signatures toward common group vocal features, resulting in group vocal signatures^34–39^. Vocal convergence is a particularly important behavior in the investigation of vocal production learning because it may have been a precursor of more complex forms of vocal production learning (e.g., vocal matching or mimicry)^17,36,39,40^.

Another powerful tool for detecting vocal production learning is quantitative genetics as it facilitates the investigation of the role of genetics and social environment in shaping vocal behavior. In vocal non-learners, kin are expected to sound more similar due to their shared genetics^41^, but in vocal learners, individuals often copy sounds from distantly related social partners, eroding that similarity^34,37,42–44^. Thus, if distantly related individuals sound more similar than their close kin, this could suggest that a form of vocal imitation occurs. Furthermore, according to the phenotypic plasticity continuum^45^, it is possible to distinguish vocal learners from vocal non-learners, by partitioning genetics from social environment and by determining how these latter factors contribute to the phenotypic plasticity and variation of vocalizations^46^. Accordingly, vocal learners are expected to have a phenotypic vocal plasticity strongly associated with social environment, while vocal non-learners are expected to show limited voluntary vocal control and display minimal phenotypic vocal plasticity associated with social environment (i.e., indicative of a stronger genetic basis of vocalizations)^45,47^.

By using alternative and integrative approaches, we aim to determine whether the rifleman (titipounamu, *Acanthisitta chloris*), one of the only two extant species of New Zealand Wrens, has predispositions for vocal production learning (e.g., rudimentary vocal learning abilities). Among riflemen’s large call repertoire^48^, feeding calls are a good candidate for this investigation. They are produced in a cooperative breeding social context by both parents, kin and unrelated helpers at nests^49,50^. Vocal learning (if present) is most likely to have evolved in such a social context, in contrast to non-interactive solitary contexts. Furthermore, rifleman feeding calls are social contact calls, which are often learned in avian vocal learners, such as in parrot and zebra finches’ contact calls and the flight calls of the Carduelinae subfamily^42,51–54^.

Here, we search for predispositions for vocal production learning in the rifleman in two ways: (1) by investigating the presence of individual and group vocal signatures and determining how genetic relatedness and social proximity influence the acoustic similarity and feeding call features of distantly related individuals living in close proximity; (2) by disentangling the genetically driven phenotypic variances of rifleman call features from their socially driven vocal counterparts using quantitative genetics, and by comparing those ratios to a vocal learner, the zebra finch (*Taeniopygia guttata*)^54^.

## Results

### Individual and group vocal signatures in riflemen

Vocal production learning can be revealed when animals copy the unique and distinctive vocalizations of another individual, known as individual vocal signatures^10,37^. Most animals that communicate with sounds, including vocal non-learners, are distinguishable thanks to their unique vocal signatures^55–60^. But, vocal learners can go one step further and learn to copy other’s unique vocal signature^37,40^. In riflemen, only weak signs of vocal individuality have been found to date in their feeding calls^61^, and it remains unknown whether they learn to copy conspecifics’ individual vocal signatures.

**Fig. 2:**
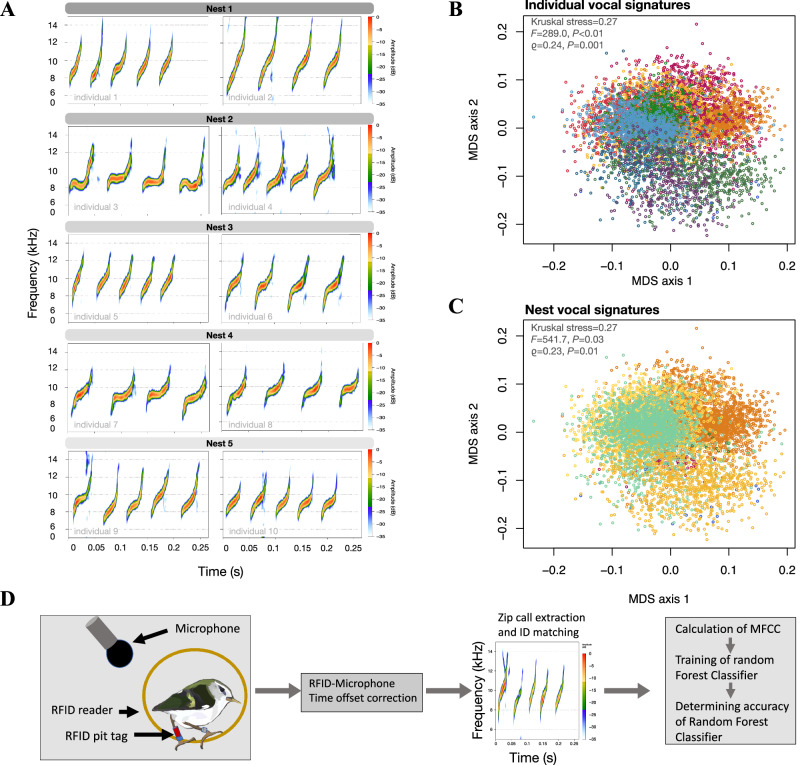
Individual and group vocal signatures in the feeding calls of riflemen. **A** Examples of rifleman feeding call spectrograms concatenated together from different time events showing unique individual vocal signatures. Concatenated calls in each frame come from a single individual. The gray colored bar above each frame groups individuals provisioning the same nest. **B**, **C** The multidimensional scaling is based on call dissimilarities of 6839 rifleman feeding calls and shows clusters of (**B**) distinct individual vocal signatures (each color represents calls from a single individual) and (**C**) group vocal signatures at the nest level (each color represents calls from individuals feeding chicks at the same nest; *n* = 6 nests, *n* = 13 individuals). **D** Training of a Random Forest Classifier resulted in the accurate classification of rifleman feeding calls to the correct individual with 82.95% accuracy and the correct nest with 85.96% accuracy.

Thanks to novel high-quality recording techniques, we detected the presence of strong individual vocal signatures in rifleman feeding calls and found that adults provisioning at the same nest were more similar to one another than random individuals. Riflemen produced visually distinctive individual vocal signatures with a strong stereotypy (i.e. structural consistency between vocalizations within an individual, Fig. 2A; *n* = 6839 calls; *n* = 13 individuals; *isoMDS Kruskal stress* = 0.27; *iterations* = 200; *k-dimensional* = 2), which were more similar within individuals than between individuals (PERMANOVA of cross-correlations: *F* = 289.9; *P* < 0.01 and Mantel *ϱ* = 0.24; *P* = 0.001; number of permutations: 10,000; Fig. 2A, B). In addition, rifleman social partners (i.e. parents and helpers provisioning the same nest) were more similar to one another than to individuals from other nests (*isoMDS Kruskal stress* = 0.27; *iterations* = 200; *k-dimensional* = 2; PERMANOVA of cross-correlations: *F* = 541.7; *P* = 0.03 and Mantel *ϱ* = 0.23; *P* = 0.001; number of permutations=10,000; Fig. 2C), revealing the presence of group vocal signatures in riflemen.

Machine learning algorithms are capable of recognizing and distinguishing individual and group vocal signatures^10,62,63^. Thus, to further confirm the presence of individual and group vocal signatures in riflemen, we trained a Random Forest machine learning algorithm^64^ with the above datasets to examine classification accuracies at the individual and nest (group) level (Fig. 2D). The Random Forest Classifier accurately classified calls to the right individual with 82.95% accuracy (*95% CI* = 0.80, 0.85; *κ* = 0.80; *P* < 2.2e-16; number of times cases are ‘out-of-bag’; computing OOB estimate of error rate: 20%, *number of trees* = 500; Fig. 2D), and to the correct nest with 85.96% accuracy (*95%CI* = 0.84, 0.88; *P* < 2.2e-16; *κ* = 0.84; computing OOB estimate of error rate: 17.5%, *number of trees* = 500; Fig. 2D). These results outperform previous vocal identification classification results for riflemen that used discriminant analysis (82.95% in this study vs. 26% Khwaja et al.^61^). Our high-quality audio recordings (with little signal to noise ratio) of rifleman feeding calls likely facilitated the distinction between individuals and groups. This result confirmed that both individual and nest vocal signatures are present, distinctive, and identifiable in riflemen.

### High acoustic similarity is not explained by genetic similarity

**Fig. 3:**
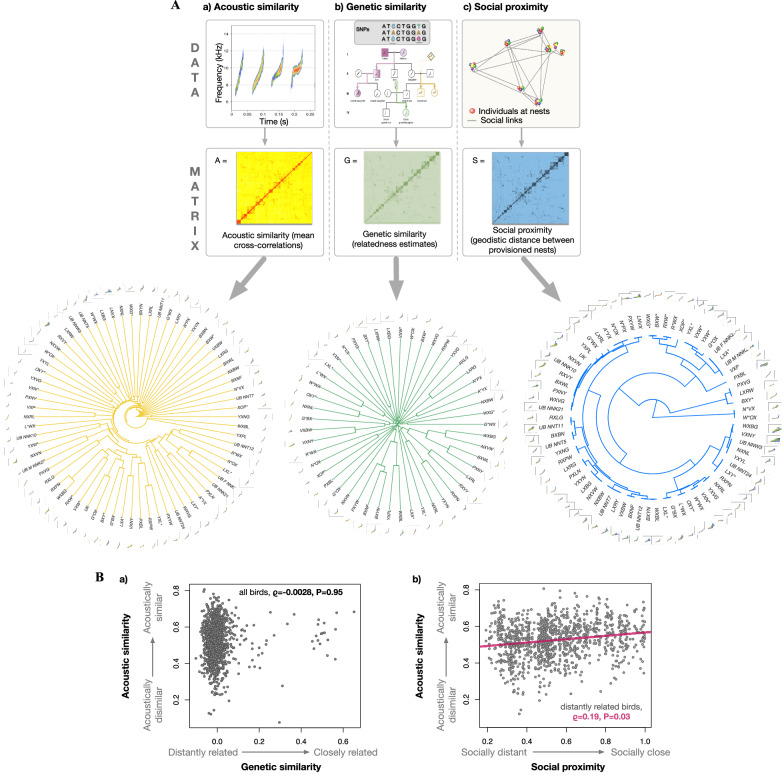
Methodology and relationship between acoustic, genetic and social similarity in rifleman feeding calls. **A** Methodology used to obtain acoustic, genetic and social similarity multiple-matrices and hierarchical-clustering phyloacoustic trees of rifleman calls. **a** The acoustic similarity matrix and the hierarchical-clustering phyloacoustic tree were derived from spectrographic cross-correlations of rifleman feeding calls (i.e. zip calls; 1110 sound clips from 70 individuals across 29 nests); (**b**) The genetic relatedness matrix was derived from SNPs data from 186 riflemen and the hierarchical-clustering phyloacoustic tree was based on genetic relatedness and acoustic data from 49 individuals; (**c**) The social proximity distance matrix and the hierarchical-clustering phyloacoustic tree were derived from geodistance proximity (i.e., based on nest locations) of social partners provisioning the same nests (*n* = 70 individuals across 29 nests). The hierarchical-clustering phyloacoustic trees have at their tips, bird identity with one representative feeding call spectrogram. The diagrams for “DATA” and “MATRIX” are for methodology illustration purposes only. **B** (**a**) Relationship between acoustic similarity (based on mean spectrographic cross-correlation of rifleman feeding calls) and genetic similarity (based on SNP relatedness estimates) (1176 bird pairs across 49 individuals). Each circle represents one bird pair. **b** Relationship between pairwise acoustic similarity (mean spectrographic cross-correlation of rifleman feeding calls) and social proximity (based on geodesic distances between nests visited by individuals) among distantly related pairs of riflemen (1149 bird pairs across 49 individuals). Each circle represents one bird pair.

We hypothesized that if distantly related riflemen sound more similar than their close kin, this could suggest that a form of vocal imitation occurs. By examining the relationship between pairwise acoustic similarity (using mean spectrographic cross-correlations^65^) and genetic relatedness in a wild population of riflemen (using 32,948 Single Nucleotide Polymorphisms or SNPs generated with Genotyping-By-Sequencing^66–68^; Fig. 3A), we found that the correlation between genetic similarity (i.e., relatedness) and acoustic similarity of feeding calls was low (Spearman’s correlation *ϱ* = 0.0028, *P* = 0.94, *n* = 49 individuals with genetic data and with a maximum of 50 randomly selected feeding calls; *npairs* = 1176 Fig. 3B.a). This indicates that genetic similarity is a poor predictor of acoustic similarity in riflemen. Furthermore, we found no correlation between genetic relatedness and mean difference in a comprehensive range of specific acoustic parameters, including call frequency and call duration (Tables S1-S2), consistent with the idea that factors other than genetic relatedness influence call similarity and vocal feature differences in riflemen.

### Socially-close, but distantly related riflemen sound similar

Vocal learners often imitate individuals that are in close proximity, regardless of their relatedness^8,69,70^. Following a similar approach to the above section, and by examining the correlations between pairwise acoustic similarity and social proximity (i.e., low mean geographic distance between individuals based on nest attendance indicates high social proximity), we found that acoustic similarity was positively correlated with social proximity in all birds (Spearman’s correlation, *ϱ* = 0.20, *P* = 0.0011, *Nperm* = 10,000, *Nindividuals* = 70, *npairs* = 2415 – note that the number of individuals *n* = 70 differs from *n* = 49 from above – see method). In other words, social proximity appears to play a crucial role in shaping rifleman call similarity.

Riflemen are facultative cooperative breeders that live within close proximity with relatives and helpers in kin-based neighborhoods^50,71^. Our results confirmed the presence of kin-based neighborhoods in riflemen and showed that relatives were socially closer than distantly related pairs of individuals (Mantel statistics Spearman’s correlation: *ϱ* = 0.15, *P* = 0.0005, *Nperm* = 10,000, *Nindividuals* = 49 birds, *npairs* = 1176; Fig. S1.a). However, this meant we could not exclude the possibility that genetic relatedness was driving the high acoustic similarity among socially close individuals. To control for this, we repeated the above analysis (i.e. acoustic similarity *vs* social proximity), but excluded pairs of close genetic relatives from our pairwise comparisons: G ≥ 0.2. The positive correlation between pairwise social proximity and acoustic similarity persisted in distantly related individuals (Spearman’s correlation: *ϱ* = 0.19, *P* = 0.032, *Nperm* = 10,000, *Nindividuals* = 49, *npairs* = 1149; Fig. 3B.b). A similar pattern was also detected among closely related individuals (excluding distantly related individuals), but although the relationship was stronger than in distantly related individuals, it was not statistically significant (*ϱ*=0.41, *P* = 0.98, *Nperm* = 1262, *Nindividuals* = 29, *npairs* = 27; Fig. S1.b). Overall, this provides additional evidence that a form of vocal convergence or possibly imitation is present in the rifleman.

**Fig. 4:**
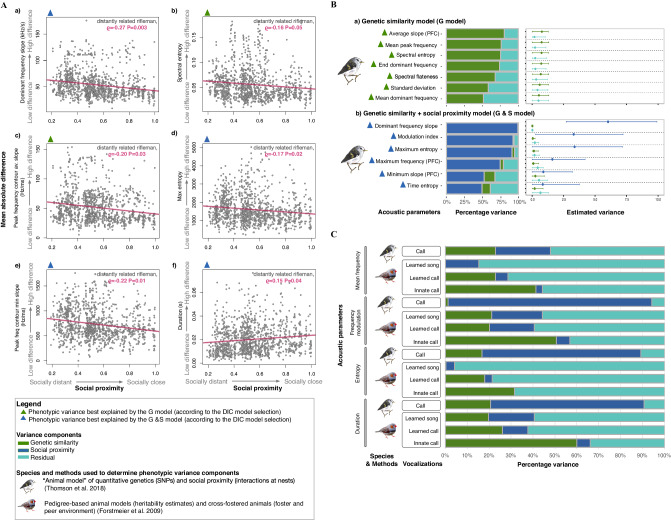
Influence of social proximity on rifleman feeding calls and comparison of phenotypic vocal variances with zebra finches. **A** Significant correlations between the mean absolute difference of acoustic parameters and social proximity among distantly related pairs of riflemen (1149 bird pairs across 49 birds). **B** Examples of phenotypic variances of selected acoustic parameters that were best explained by: a) the genetic similarity model (G model; *n* = 39 individuals), and b) the genetic similarity and social proximity model (G & S model; *n* = 39 individuals). The complete set of comparisons for each model can be found in Figs. S4–S6. The social proximity animal model (S model) is not represented because none of the acoustic parameter variances could be explained by this model (Fig. S5). **C** Comparison between the phenotypic vocal variances of rifleman feeding calls, zebra finch females’ innate calls, and zebra finch males’ learned calls and songs. Two different methods and models were used to determine phenotypic variances in riflemen and zebra finches^46,54^. For best comparison, the social components (i.e., peer and foster effects) in zebra finches were combined and the maternal effects were not represented.

To understand which aspects of rifleman vocalizations were adjusted in response to social proximity, we further examined the relationship between acoustic parameters of feeding calls and social proximity among distantly related individuals (Fig. 4A, Table S3). Acoustic parameter-specific Mantel tests revealed statistically significant correlations for 7 out of 37 acoustic parameters (at a two-sided significance threshold of 0.05) (Table S3). These parameters were related to frequency, such as frequency slope of feeding calls (i.e., the change in frequency through time), duration, entropy and inflections (Table S3). These correlations ranged from *ϱ* = −0.27 (*P* = 0.003) to *ϱ* = 0.20 (*P* = 0.01), with dominant frequency slope (dfslope) and minimum frequency contour slope (PFC) having the strongest correlations, and frequency median having the weakest correlations (Table S3). After accounting for multiple comparisons and correlations between acoustic parameters, the chance of obtaining a significant correlation between at least 7 acoustic parameters (out of 37 parameters) and social proximity was 0.03. In other words, it is very unlikely that one would see this many significant correlations just by chance. This suggests that some parameters are biologically relevant to rifleman social interactions. It is also worth noting that, despite some correlations being significant, the values of *ϱ* were generally low, suggesting that the correlations observed may result from subtle call feature modulations. Overall, these results show that social proximity plays a role in shaping some aspects of rifleman calls, and that individuals provisioning for the same nests share higher acoustic similarity, regardless of their genetic relatedness.

### The phenotypic variance of the rifleman calls resembles those of a vocal learner

Vocal learners are expected to have higher vocal plasticity and phenotypic vocal variances than vocal non-learners^45,54^. If riflemen vocally learn, their vocal plasticity and phenotypic vocal variance should be similar to those of vocal learners. To test this hypothesis, we built three multiple-matrix “animal models” (i.e., Genetic similarity model; Social proximity model; and Genetic similarity & Social proximity model)^46^ and used model selection with Deviance Information Criterion (DIC; Table S5) to determine which of these three models best predict the proportion of phenotypic variance components for each acoustic parameter^46^ (Fig. 4A, B).

Based on the DIC model selection, the genetic similarity model (G model) best explained the phenotypic variance of 16 out of 37 feeding calls’ acoustic parameters (Fig. 4B, Fig. S4 and Table S5). For example, the phenotypic variance of the average slope of the peak frequency contour was best explained by this model and had the strongest genetic influence (i.e., largest genetic proportion of variance and smallest residual variance). In addition, the credible interval ranged from < 0.0001 to 1.3 (Fig. 4B.a; Fig. S4). These results indicate that some acoustic parameters have a stronger genetic basis with a minimal influence by social environment.

The social proximity model (S model), on its own, did not explain the phenotypic variances of any acoustic parameters (Fig. S5; DIC Table S5). In this model, the credible interval ranged from 0 to 11.3 (Fig. S5). This result aligns with our expectations that some acoustic parameters have a strong genetic component (i.e., morphology and auditory innate vocal templates^72^), and that the social environment alone cannot contribute to the entirety of the phenotypic variance of calls.

However, the combined genetic and social model (G&S model) best explained the phenotypic variance of 21 out of 37 acoustic parameters (Fig. 4B.b; Fig. S6), according to the DIC model selection (Table S5). In this model, social variance had the largest credible intervals compared to genetic variance (range < 0.0001 to 9.9; Fig. S6). The social variance of the dominant frequency slope and the peak frequency contours of the average slope of calls had non-overlapping credible intervals which diverged away from zero, supportive of the effect of the social proximity on these parameters. This result indicates that the phenotypic variance of rifleman call features is influenced by both genetic similarity and social proximity, and that some acoustic parameters are more influenced by social proximity than genetic similarity, as would be expected in vocal learners. However, as will be discussed below, non-genetic variation may also be caused by other factors, such as shared environmental or social conditions.

## Discussion

By combining diverse quantitative approaches, we demonstrate that New Zealand wrens have unique individual vocal signatures that converge toward common vocal features among distantly related individuals that share high social proximity. This result reveals the presence of vocal convergence in riflemen. Further, we show that the phenotypic variance ratios of rifleman calls are most similar to vocal learners. These results align with the vocal learning continuum/module hypotheses, and suggest that riflemen may possess rudimentary vocal learning abilities.

Detecting whether and how individuals match each other’s unique vocal features (i.e. vocal signatures^73–75^) is one of the first stages in the investigation of vocal production learning^56,76–78^. In our study, each individual had unique and distinctive vocal signatures with high stereotypy (i.e., structural consistency between vocalizations within an individual), possibly due to individual differences in the morphology of their vocal tract (e.g. bill, syrinx size). Individual vocal signatures in social species, such as the rifleman, are beneficial because they may help rifleman parents distinguish among the multiple helpers that visit nests simultaneously^49^ .This is true especially in the context of “pay-to-stay” situations commonly found in cooperative breeding systems, in which helpers support parents in order to be accepted and tolerated within the group territory^79^. These unique individual vocal signatures may also help riflemen distinguish their breeding partner from others and may benefit parent-offspring interactions by helping nestlings and fledglings recognize and locate their parents. Although the goal of our study was not to investigate call differences within individual call repertoire, it is interesting to note the small variations within an individual’s call repertoire. This is relevant to our search as it may be indicative of syringeal or bill control.

In the context of vocal learning, animals that copy each other’s unique individual vocal signatures provide a strong indication for vocal production learning. Animals that imitate or converge toward others’ individual vocal signatures result in group vocal signatures or “vocal passwords” or accents^80,81^. This may help animals gain considerable social advantages, such as stronger bonding and group membership^35,56,70,82,83^. In species that learn their vocalizations, such as in many birds^44,82,84^, pinnipeds^3^, cetaceans^37,85,86^, bats^80^ and humans, group vocal signatures are obtained either through a process called vocal matching – an exact copy of individual vocal signatures from the same group^87^, or vocal convergence – a form of vocal imitation in which individuals slowly modify their call features toward common group vocal features (e.g., when pairing with a partner or assimilating to a new group)^70,82,83^. Vocal matching was not demonstrated in this study, but we detected vocal convergence in socially and geographically close riflemen that were provisioning for the same nest, but were not closely related. This suggests that individuals have some degree of vocal control with their vocalizations. This result contrasts with the traditional assumptions that rifleman calls have relatively little vocal plasticity and genetically encoded vocalizations.

According to Janik et al.^88^, such vocal convergence toward a common group vocal signature is a strong indicator for vocal learning ability. But recent work has also shown that vocal convergence is present in animals traditionally labeled as vocal non-learners, making it ambiguous whether vocal convergence is the result of vocal production learning^36,76,89–92^. For example, goitred gazelles (*Gazella subgutturosa*)^93^, domestic goats (*Capra hircus*)^94^, pygmy marmosets (*Cebuella pygmaea*)^95,96^ and orangutans (*Pongo sp*.)^97,98^ show high levels of vocal plasticity and modify their vocalizations toward the individual signatures of their partners or group members. Similarly, young agile gibbons (*Hylobates agilis agilis*) produce strong innate vocal templates from birth, but modulate and refine their vocalizations during ontogeny to match their mothers’ calls and songs^99^. Whether vocal convergence is learned in these animals, traditionally known as vocal non-learners, is not well understood or thoroughly tested. One possible hypothesis is that vocal convergence is expressed along a learning continuum with non-learned, rudimentary and learned forms. For example, in some species, vocal convergence may be attributable to reward-based operant conditioning^17^, which may not require cortical involvement of vocal control and may only need the lower brain region (for example, midbrain thalamic pathway) for neuromuscular modulation of simple call matching. Thus, while convergence may be the result of simple learning in some species, it may have an entirely different mechanism in other species. Further research is needed to test this hypothesis and to investigate the mechanisms underlying vocal convergence in multiple and diverse species.

Importantly, unlike vocal non-learners that vocally converge as an immediate response to a conspecific call (e.g. frequency matching in frogs^100^), rifleman vocal convergence was maintained even in the absence of other conspecifics at the nests. This indicates that the observed vocal convergence in riflemen is not a simple one-time matching or a short-term frequency shift response. This type of sustained vocal convergence has previously been observed in vocal learners such as parrots^34,44^, oscines^42^, and hummingbirds^82^, as well as in species traditionally termed vocal non-learners^76,92^, including suboscines^101^ (Fig. 1), but never in New Zealand wrens. This may suggest that the vocal convergence detected in riflemen is more similar to that of vocal learners than vocal non-learners.

Our quantitative genetic analyses further helped clarify whether learning could cause the observed vocal convergence in the rifleman. Similar to vocal learners – which generally have higher phenotypic plasticity than vocal non-learners due to their strong social influence (see phenotypic plasticity continuum by Mesoudi et al.^45^) – the rifleman had call phenotypic variances that were best explained by the combined genetic and social model, and had high proportions of social variance. Vocal production learning is one potential mechanism that could explain these results; however, some interpretative caution is needed because factors such as shared physiological and motivational parameters^102^ may have also affected call phenotypic variance. However, in our study, environmental factors, such as shared food resources or habitat differences, can be excluded because the birds shared the same general habitat. Furthermore, it is important to highlight that innate vocal behaviors produced in the total absence of vocal learning can also show strong levels of vocal plasticity under the influence of social environment (although considerably less than in vocal learners)^103^. For example, white-lipped frogs (*Leptosactylus albilabris*) are vocal non-learners that perform complex, plastic, yet instinctive vocal behaviors that match the frequency of conspecific calls^100^. But, unlike white-lipped frogs’ phenotypic vocal variance, which is predicted to be best explained by genetics (as would be expected in vocal non-learners)^104^, rifleman call phenotypic ratios were more similar to that of vocal learners (in addition to being variable and sustained over a long time period). This may indicate that their vocal plasticity is closer to that of vocal learners (with their ability to both instantaneously match a sound as well as maintain a learned sound overtime).

Comparing the phenotypic vocal variances between riflemen and zebra finches^46,54^ (Fig. 4C) helped further illustrate how rifleman’s vocal plasticity fitted in the phenotypic plasticity continuum^45^. Although differing comparative methods and models were used for this comparison, the relative genetic versus social ratios of our models were similar to that of zebra finches’ learned calls and songs (i.e., produced by males; Fig. 4C). Riflemen had an even larger social versus genetic variance ratio than zebra finches in some parameters. This further indicates that the mechanism underlying the observed variance in the rifleman calls may be closer to that of vocal learners. Conducting an extensive cross-species comparative analysis of phenotypic vocal variance components would be a valuable future comparison and contribution. Ideally, such future studies should use a single and standardized comparative method, such as using MCMCglmm of phenotypic variances between species^46^. This would further situate rifleman phenotypic variances relative to other vocal non-learners and vocal learners along a phenotypic plasticity continuum (Mesoudi et al.^45^).

In conclusion, this study offers new research avenues and methodologies to investigate potential predispositions for vocal learning abilities in presumed vocal non-learners. It reveals the presence of vocal convergence and possibly a rudimentary form of vocal learning in the rifleman. This raises important questions about the evolution of vocal convergence, which might have been present in the shared ancestor of Psittacopasserans. Vocal convergence and any predispositions for vocal production learning are behaviors that are easily overlooked without extensive analyses, so sophisticated and in-depth future studies are needed to explore their possible existence and their origins in other animal groups. In particular, exploring the vocal phenotypic variances of traditional vocal non-learners would provide an important contrast to our findings. It would also provide a framework for others trying to interpret phenotypic variances in the context of the vocal learning continuum hypothesis. Further, our study highlights the importance of revisiting the definition of vocal convergence in the context of vocal learning. Overall, this work offers new insights into the vocal behavior and learning predispositions of a presumed vocal non-learner, the rifleman – a species which is key to understanding the evolutionary origin of vocal production learning in Passeriformes.

## Methods

### Study field site and population monitoring

We monitored a wild population of riflemen at the Boundary Stream Mainland Island reserve, New Zealand (39°06’15.8“S, 176°48’06.1“E) during two austral breeding seasons, from September to February 2018–2020. The timing of rifleman breeding is asynchronous^25^, which enabled us to monitor nests simultaneously and continuously throughout the breeding seasons. For monitoring and identification purposes, we caught individuals using mist-nets and speakers with conspecific lure calls, we then sexed and aged adults and fledglings based on their sexual dimorphism in size and coloration^105^, and we banded individuals with unique color band combinations. One of the leg bands was equipped with a Passive Integrated Transponder (2.3 mm EM4102 PIT tag; Eccel Technology Embedded RFID) that could be read by Radio-frequency identification receivers (RFID; engineered by the University of Auckland) placed at nests (Fig. 2D). RFID receivers logged the identity of individuals entering or leaving nests during the 2019-2020 breeding season. After locating nests, we monitored nest activity and recorded vocal behavior of individuals provisioning nests (see recording methods below). Due to the inaccessibility of most natural nests (i.e., tight spherical nests in tree cavities), we could not band nestlings which limited information about the relatedness among individuals. To mitigate this, we banded fledgling groups when siblings still clustered together outside their nests and were fed by banded adults.

We have complied with all relevant ethical regulations for animal use. This research was approved and facilitated by mana whenua from the Maungaharuru region and the Department of Conservation, New Zealand (Department of Conservation permit obtained in 2018, act No FAU 55391) and approved by the University of Auckland Animal Ethics Committee (Approval no. 001866). Bird capture and banding activities were conducted under the New Zealand National Bird Banding Scheme.

### Study species

Riflemen are socially monogamous cavity-nesters that build their nests at various heights (i.e., on the ground under leaf litter or in tree cavities) in the native forests of New Zealand^25^. Nest building can last a few days (i.e., 3–6 days, personal observation) and is followed by an incubation period which can last 20 days^25^. Once hatched, nestlings remain in the nest for around 24 days^25^. Riflemen are facultative cooperative breeders (i.e., helpers in addition to parents feed nestlings at some nests), so nestlings receive frequent visits from both parents and helpers which are often genetically related to the parents^49,50,71,106^. The frequency of visits at the nests increases as nestlings grow, with a feeding rate ranging from 4 to 20 times an hour (i.e., every 3 to 12 min) in the later nestling stages^25^. Each time adults visit nestlings, they produce feeding calls (also known as zip calls; e.g. Fig. 2A) prior to feeding them which seems to function as contact calls between parents and offspring^25^. These feeding calls are distinguishable from other call types due to their “S” shape and higher frequency (*~*6–14 kHz; Fig. 2A)^48^. Rifleman feeding calls are a good candidate to test the presence of vocal learning abilities because they are used in social contexts (between parents, helpers and nestlings, and between partners) in which vocal learning is most likely to have evolved and be detected if present. The rifleman does not produce vocalizations that classify as songs in the traditional sense; indeed, it is a non-territorial species and rifleman pairs do not seem to produce courtship songs to attract mates. Instead, the rifleman has a large repertoire of calls^48^, which has been assumed to be innate based on its simple vocal features, nonterritorial or non-courtship vocalizations, and a syrinx that lacks complex intrinsic muscles found in bird vocal learners^21,22,24,25^.

### Sound recording

We collected rifleman feeding calls (zip calls; e.g. Fig. 2A) at nests using a combination of methods which we implemented to accommodate the diversity of nest heights found in our study population. First, we collected focal recordings of the feeding calls at nest (i.e. one observer with binoculars, a digital Zoom H6 recorder and a shotgun Sennheiser ME62 K6 microphone -20,000 Hz frequency) of banded individuals (i.e. identified by their unique color band combo). During focal monitoring sessions, we combined focal recordings with visual monitoring and video recordings to match the identity of individuals visiting nests to their corresponding feeding calls. We also recorded the vocal behavior of individuals at nests using passive Bioacoustic Automated Recorders (BAR; Frontier Labs version 1.4; WAV format with a sampling frequency of 44,100 Hz and 32 bits sampling depth; breeding season 20182020; Fig. 2D). Each BAR recorder was connected via a long cable to a small omnidirectional microphone placed close to nest entrances (range: 0.1 m–1 m). Each BAR recorder was placed further away from the focal nest (10–15 meters) to minimize nest disturbance when changing batteries and SD cards. BARs were programmed to record daily from 1 hour before sunrise to 2 h after sunset. In addition to the BAR recorders, we also deployed trail cameras (Bushnell Trophy Cameras 24MP and E2 12MP) and PIT tag readers (2.3 mm EM4102 PIT tag; Eccel Technology Embedded RFID; breeding season 2019–2020) to facilitate the synchronization of individual identity and feeding calls. The trail cameras pointed toward nest entrances (i.e., placed between ~30 cm and ~1 m) to capture photos and videos of the leg color bands combinations, and PIT tag readers were placed around nest entrances to log PIT tag numbers.

### Sound processing and annotations

#### Time offset correction and synchronization

Time drift occurred in some of the BAR recordings due to SD card write-speed differences, which resulted in a mismatch between the timestamps of the BAR recorder and RFID (Fig. 2D). We corrected for time offset using known sound timestamps produced in the BAR recordings for which we had an exact RFID timestamp. For both the time offset correction and the following synchronization step, we used the following custom pipeline (available at https://uoa-eresearch.github.io/bird_recognition/time_sync_check.nb.html). The synchronization between the timestamps of PIT tags and nest recordings enabled us to associate calls to the correct individuals. We extracted feeding calls (zip calls; e.g.: Fig. 2A) based on PIT tag readings of individuals entering nests within 5 s of a PIT tag time-stamp reading. Samples with two or more individuals detected within these 5 seconds were removed to avoid errors in identity attribution. We then manually annotated each feeding call within these 5-second filtered time windows using Raven Pro v1.6.1^107^.

#### Sound libraries

The above sound collection method yielded a large and high-quality RFID/BAR call library with 6839 calls from 13 individuals (12 parents and one nest with one helper; *mean* = 526.1 *± sd* = 456.4 calls per individual, *min* = 12 calls, *max* = 1378 calls) across 6 nests (*mean* = 977.0 *± sd* = 1,012.8 calls, *min* = 12 calls, *max* = 2457 calls). This library offered a large number of calls per individual and high-quality sound clips with no background or overlapping sounds which is ideal to detect vocal signatures and train our machine learning algorithm (Fig. 2).

We also manually synchronized the BAR or focal recordings to the timestamps of our visual observations, trail cameras, and video recording timestamps using Raven Pro v1.6.1^107^. This combination of recording techniques (BAR/focal/trail camera/video) enabled us to build a second extensive feeding call library with 4207 calls from 70 birds across 29 nests (see details below under “*Creation of acoustic, genetic relatedness, and social matrices*”). We used this call library and the above RFID/BAR call library for the remaining analysis of our study (Figs. 3–4).

#### Spectrograms and call feature measurements

All spectrograms of feeding calls were plotted with *Seewave* v.2.2.0^108^ (settings set to wl=250, ovlp=50) and *warbleR* v.1.1.27^65^ (Figs. 2A, 3A respectively). We measured feeding call features using three vocal measurement tools (*Seewave* v.2.2.0^108^, *warbleR* v.1.1.27^65^, and RavenPro *Raven Pro* v.1.6.1^107^), and extracted 37 acoustic parameters (Table S1).

### Detection of vocal signatures

To assess the presence of vocal signatures at the individual and nest level in rifleman, we compared feeding calls within and between individuals attending the same nest. We performed spectrographic cross-correlations between the feeding calls of each individual, which is a method that slides spectrograms over one another to obtain a similarity score between calls (i.e., similarity matrix, based on sound dissimilarities – one minus cross-correlations rescaled to be between 0 and 1). We used the R function xcorr from *warbleR* v.1.1.26^65^ to perform the pairwise cross-correlations (Fig. 2B, C). All pairwise combinations of calls resulted in 6, 839 *×* (6, 839 − 1)*/*2 distinct cross-correlations of calls for 13 individuals (*mean* = 526.1 *± sd* = 456.4 calls per individual; *min* = 12 calls, *max* = 1378 calls) across 6 nests (*mean* = 977.0 *± sd* = 1012.8 calls; *min* = 12 calls, *max* = 2457 calls).

We then compared the pairwise cross-correlations using Kruskal’s non-metric multidimensional scaling with *MASS* (isoMDS) v.7.3.51.6^109^ (Fig. 2B, C), and analyzed the goodness of fit with isoMDS Kruskal’s stress^109^. We then used a Mantel test based on Pearson’s correlation from *vegan* v.2.5.6^110^ to determine whether vocal signature was significant at the individual or nest level. To do this, we created a binary matrix representing feeding call membership in which 0 was assigned to feeding calls belonging to the same individual and 1 was assigned to feeding calls belonging to different individuals^82^. The cross-correlation dissimilarity matrix (1-correlation similarity matrix) was then compared with the membership matrix using a Mantel test, and it used group membership matrices as predictors in Mantel correlations against acoustic dissimilarity matrices. We also used PERMANOVA^111^ (adonis from *vegan* v.2.5.6^110^) as an alternative method to detect individuality in the feeding calls of riflemen. For nest signatures, we controlled for individual vocal signatures (i.e., by only permuting individuals, rather than clips between individuals) because individual vocal signatures could otherwise cause an apparent nest vocal signature due to the small number of individuals per nest.

### Random forest classifier

We created and trained a Random Forest Classifier using calls collected from our BAR/RFID library (Fig. 2A) to test if the classification of feeding calls could be accurately achieved at the individual and group level (Fig. 2D). We normalized feeding calls at -1dB using *ffmpeg* v1.20.1^112^ to ensure that amplitude differences between recording clips would not cause classification inaccuracies. We compiled the training library by loading .wav files of feeding calls and then calculated Mel-frequency Cepstral Coefficients (MFCC) of the feeding calls using *tuneR* (melfcc) v1.3.2^113^. MFCC have been extensively used for human voice recognition^114^ and focus on dynamic features of the vocal tract, and have been successfully applied to animal vocalization recognition^115^. We then set up the duration of each time frame (i.e., used as a “sample”) at 0.25 s. A longer duration meant that more call clips were included in one “sample”, thus making the classification easier; however, it also meant that from each particular .wav file, fewer “samples” could be extracted. Consequently, we set hoptime to be the same as wintime, so that successive time frames could not overlap, making the samples truly independent. Next, we randomized the order of time frames (e.g., in case bird clips had not been concatenated randomly). We then trained a Random Forest Classifier, which used multiple learning algorithms (ensemble learning) to obtain high predictive performance for classification purposes, using the function random Forest from *randomForest* v.4.6.14^64^. We then estimated the accuracy of the classifier with the samples from each group (individual and nest).

### Blood sample collection and DNA extraction

We collected blood samples (10–35 µl) during banding sessions using brachial venipuncture with a BD PrecisionGlide sterile needle (26 G 1/2–0.45 mm x 13 mm) and a 70 µl capillary tube (Microhematocrit tubes). Blood samples were preserved in 95% ethanol and temporarily stored at 4 °C in the field before being transferred for long-term storage at −20 °C. In addition to collecting blood samples from Boundary Stream Mainland Island (i.e., focal population), we also collected blood samples from Mohi Bush (39°51'25.34"S, 176°54'7.49"E), a geographically close but genetically distant population to increase our sample size to 186 individuals to provide robust genetic relatedness estimates^116^. We extracted DNA from individual blood samples using a Qiagen DNeasy Blood and Tissue kit and followed the protocol for total DNA purification from nucleated blood with elution in 80 µl AE buffer and without RNase treatment (Spin-column Protocol; DNeasy Blood & Tissue Handbook version 07/2006). DNA was quantified using the Qubit Broad Range DNA assay (Qubit 2.0 Fluorometer), and further quality checks were performed by spectrophotometry (Implen NanoPhotometer N60) and by gel electrophoresis. DNA aliquots were dried (3 h. at 30 °C) and sealed for shipping to AgResearch, Mosgiel, Aotearoa, New Zealand, where a Genotyping-By-Sequencing (GBS) protocol was carried out.

### GBS library construction and SNPs detection

High-throughput Genotyping-By-Sequencing (GBS) was used to generate Single Nucleotide Polymorphisms (SNPs). The GBS library was prepared following the method outlined in Elshire et al.^66^ with modification as in Dodds et al.^67^ (*KGD* v0.8.4) for 186 individuals (total of 192 wells with 6 negative control samples) using *PstI-MspI* double-digest restriction enzymes. The Library underwent size selection with Pippin Prep (SAGE Science, Beverly, Massachusetts, USA) to select fragments in the size range of 193−318 bp (genomic sequence plus 123 bp of adapters). Single-end sequencing (1x101bp) was performed on an Illumina HiSeq 2500 utilizing v4 chemistry. Raw fastq files were quality checked using a custom QC pipeline (available at https://github.com/AgResearch/DECONVQC and *FastQC* v.0.10.1, http://www.bioinformatics.babraham.ac.uk/projects/fastqc/, Andrew^117^).

Demultiplexing, SNP detection, and filtering were undertaken with the reference-free SNP detection pipeline called *UNEAK*^118^, implemented in *Tassel3* v.3.0.174^119^. We filtered SNPs to avoid the inclusion of erroneous SNPs in downstream analyses. The following parameters were used: s (maximum number of barcoded reads per lane) set to 400 M, t (merge taxa option) set to “no”, m (maximum tag number in the merged TagCount file) set to 600 M, x (Maximum tag number in TagCount file for each taxa) set to 100 M, c (minimum count of a tag that must be present to be output) set to 30, mnMAF (minimum minor allele frequency) set to 0.03, and mnC (minimum call rate) set to 0.1 (10%). All other parameters (e.g., Error tolerance rate – ETR; Maximum minor allele frequency –mxMAF; maximum call rate – mxC) were left at the default option (i.e., ETR = 0.03; mxMAF=0.5; mxC=1). We excluded SNPS with a significant (*p* < 0.05) deviation from Hardy-Weinberg equilibrium (Fig. S2). We identified 32,948 SNPs (*mean co-call rate for sample pairs* = 0.55; *min co-call rate for sample pairs* = 0.23; *proportion of missing genotypes* = 0.36; *call rate* = 0.64) and the average sequencing read depth for called SNPs was 7.6.

### Creation of acoustic, genetic relatedness, and social matrices

To further investigate the mechanisms resulting in nest vocal signatures, we created three matrices (i.e., acoustic matrix, genetic relatedness, and social proximity matrices; Fig. 3A) for downstream analyses to disentangle the genetic and social factors influencing rifleman call structure (i.e., similarity). In vocal learners, social factors influence acoustic structure and similarity between individuals, especially in individuals that are not closely related, so these analyses can help detect predispositions for vocal imitation or learning abilities^45,120^.

First, we determined acoustic similarities between individuals by creating an acoustic matrix for all individuals for which we had collected call clips (70 birds across 29 nests; Fig. 3A.a). Some individuals had considerably fewer feeding calls than others due to rare nest visitations (e.g., helpers) or limited available recordings, which resulted in an imbalanced dataset. Thus, to balance out our dataset of calls per individual, we randomly selected a maximum of 50 feeding calls per individual. This resulted in a total of 1,110 filtered sound clips (*mean* = 14.4 calls per individual, *sd* = 14.3 calls, *min* = 1, *max* = 50). We then calculated the mean of pairwise spectrographic cross-correlations between rifleman feeding calls using the function *xcorr* from *warbleR* v.1.1.27^65^. The *warblerR* settings for xcorr were set as follows: window length (*wl*) = 300 and overlap (*ovlp*) = 90. All pairwise combinations of calls resulted in 1110 × 1110 distinct cross-correlations of calls and 70 × 70 distinct mean cross-correlations for individuals.

Next, following the KGD v0.8.4 pipeline (G5 method, as in Dodds et al.^67,68^), we determined the genetic relatedness between individuals using the 32,948 filtered SNPs (obtained from the above method) to generate a genetic relatedness matrix (GRM) and to measure genetic similarity (or genetic distance) between individuals (*n* = 186 individuals, Fig. 3A.b). Genetic relatedness between individuals was visualized using a relatedness heatmap (Fig. S2). It is important to note that genetic relatedness was determined here, not the heritability of any vocal trait.

Finally, to create a social proximity matrix that reflected social closeness between individuals, each individual for which we had acoustic data (*n* = 70 birds across *n* = 29 nests) was given a location (GPS points) based on the location of the nest they provisioned (Fig. 3A.c). Because several adults attended the same nest, we “blurred/jittered” the GPS points to a few centimeters away from the commonly shared GPS nest point to avoid having all individuals provisioning for a same nest with a social distance of 0. This “blurring/jittering” was essential to retain a true social proximity matrix that could become invertible (i.e., positive definite), as recommended by Thomson et al.^46^. We used the package *castor* v.1.7.2^121^ and the function all_pairwise_geodesic_angles to calculate the distance between two sets of individual locations/nests coordinates. This function returns a 2D matrix of size N1 x N2 (in this case, 70*70). If one individual was found at several nests, we calculated the average distance for that individual between all provisioned nests. We used this distance as a proxy for a shared social environment (i.e., social interactions) to generate a social proximity matrix (Fig. 3A.c).

### Hierarchical-clustering trees with spectrograms

We visualized call similarity between individuals by generating hierarchical-clustering trees with spectrograms and individual identity at their tips (Fig. 3A), based on acoustic, genetic and social proximity distances between individuals (based on GPS locations of individuals provisioning at nests) using the package *ape* v.5.6-2^122^ and phylo_spectro from *warbleR* v.1.1.27^65^. One randomly selected feeding call from each individual call repertoire was represented as a spectrogram at the tips of the trees’ branches. The *warbleR* settings for the phylo_spectro function were set as follows: *wl* = 300, *ovlp* = 90, *wl.freq* = 512.

### Correlations between acoustic, genetic, and social similarity

To determine whether and how the social environment and genetics contribute to shaping rifleman nest vocal signatures and whether this could reveal any predispositions for vocal learning (e.g. high acoustic similarity among distantly related pairs of individuals), we examined the relationship between the acoustic (i.e., mean spectrographic cross-correlation of calls per bird), genetic (i.e., SNP relatedness estimates) and social similarity (geodesic distances between nests provisioned by individuals) (Fig. 3; following similar reasoning as in Lemasson et al.^120^).

To assess the correlations between these matrices, we used Mantel tests (Spearman’s correlation with permutation null model; two-sided significance) with the function *cor* from the R package *stats* v4.0.2^123^ (Figs. 3B, 4A; Tables S2, S3). Note that when comparing pairwise distances, the individual pairs cannot be considered independent samples because different pairs may correspond to shared individuals, hence the statistical significance of any given correlation is generally estimated using permutation tests that account for this data structure^124,125^.

First, we used a Mantel test (Spearman’s correlation with permutation null model; two-sided significance) to examine the relationship (i.e., correlation) between genetic similarity and acoustic similarity (1176 bird pairs across 49 birds; Fig. 3B.a). 49 individuals (out of 186 individuals for which we had genetic data) had both genetic relatedness and acoustic data. The mean spectrographic cross-correlation for these 49 individuals was based on 929 sound clips (*mean* = 19.0 calls per bird, *sd* = 15.4). We expected that if acoustic and genetic similarity were strongly correlated, this would indicate that genetics has likely a strong influence on acoustic similarity. In contrast a weak or negative correlation would indicate that other factors shape acoustic similarity. For this analysis, we excluded self-related acoustic similarity from our analysis because the “within-individual” vocal signatures would have otherwise biased the correlations between the two entries.

Next, we used a similar Mantel test as above to examine the relationship between acoustic similarity and social proximity and the relationship between social similarity and genetic similarity (Fig. S1a). This enabled us to determine whether individuals living in close proximity sounded similar and whether they were kin (i.e., kin-neighborhoods). After confirming the influence of social proximity on acoustic similarity in all birds (Spearman’s correlation, ϱ = 0.20, *P* = 0.0011, Nperm = 10,000, Nindividuals = 70, npairs = 2415) and detecting kin-neighborhood effect in rifleman (Spearman’s correlation: ϱ = 0.15, *P* = 0.0005, Nperm=10,000, Nindividuals = 49 birds, npairs = 1176, Fig. S1a), we controlled for genetic relatedness, by re-examining the relationship between acoustic similarity and social proximity, but this time we restricted our analysis to distantly related pairs of individuals by excluding genetically close pairs of individuals (Fig. 3B.b). To exclude genetically close pairs of individuals we set a maximum relatedness threshold to 0.2 (i.e., excluding siblings, parents, uncles, and aunts from the genetic matrix and the correlation calculations). Among those 1176 bird pairs for which genetic relatedness was known, 1149 bird pairs had a genetic similarity below 0.2 (i.e., “distantly related” or “unrelated”), and the remaining 27 pairs had a genetic similarity above 0.2 (i.e., they are “closely related”). The 1149 distantly related pairs covered all 49 birds, in other words for every bird in our dataset there exists another bird that is unrelated to it. The 27 closely related pairs cover 29 individuals, in other words there are 29 birds in our dataset for which there exists another closely related bird.

Next, to determine which specific acoustic parameters were driving the significant positive correlation between acoustic similarity and social proximity among distantly related pairs of individuals (Fig. 3B.b), we investigated the relationship between the mean absolute difference in 37 acoustic parameters between any two sound clips of two individuals and social proximity in distantly related pairs of birds (1149 across 49 individuals, Fig. 4A and Table S3). The *warbleR*v.1.1.27 function specan^65^ and *Raven Pro* v.1.6.1^107^ were used to measure the 37 acoustic parameters of rifleman feeding calls (see list of the acoustic parameters and their descriptions can be found in Table S1). The *warbleR* settings for the specan function were set as follows: *wl* = 300, *ovlp* = 90, *wl.freq* = 512. We then used acoustic-parameter-specific Mantel tests (two-sided significance threshold of 0.05) to examine correlations between mean absolute acoustic difference and genetic similarity (Table S2), and mean absolute acoustic difference and social proximity for each acoustic parameter (Fig. 4A; Table S3). Acoustic-parameter-specific Mantel tests revealed statistically significant correlations with social proximity for 7 out of 37 vocal parameters (at a two-sided significance threshold of 0.05; Table S3). Mutual pairwise spearman correlations showed these latter parameters were independent of each other (Table S4). To account for multiple comparisons and examine how probable it would be to erroneously obtain at least this many significant correlations by chance (i.e., under the null hypothesis of no correlations with social proximity), we used an adjusted permutation test. Our test fully accounted for correlations between acoustic parameters. Specifically, rows and columns of the social distance matrix were synchronously permuted (to break any association with acoustic parameters, as in a regular Mantel test), and subsequently all 37 acoustic-parameter-specific Mantel tests were repeated to re-compute the number of significant correlations. This permutation step was repeated 1000 times, to obtain the fraction of permutations that yielded at least 7 significant correlations. This fraction is an estimate for the probability of erroneously seeing a significant correlation between at least 7 acoustic parameters and social proximity, under the null hypothesis. We found that this probability was 0.03.

### Multiple-matrix animal models: Estimation of the phenotypic vocal variance of rifleman feeding calls

Next, we built multiple-matrix animal models to partition the phenotypic variance of the rifleman feeding calls for each acoustic parameter (Fig. 4B, C). Models with strong social influence (i.e., acoustic parameters with high social variance components) are more likely to indicate the presence of social learning possibly via a vocal production learning mechanism^45^. We followed methods from Thomson et al.^46^, which uses Markov Chain Bayesian generalized linear mixed effect models (MCMCglmm) – an approach that fits an animal model into a Bayesian framework (Fig. 4B, C) to estimate acoustic traits’ genetic, social, and residual variance components for each acoustic parameter. We used *MCMCglmm* v.2.34^126^ and built generalized linear mixed effect models (GLMM) with the previously generated genetic, and social matrices (Fig. 3A). For all three models, acoustic traits were continuous traits added as fixed effects.

The MCMC ran for *n* = 1,000,000 iterations with thinning interval (*n* = 100), burn-in period (*n* = 100,000) for each acoustic parameter. We used *n* = 1,000,000 iterations to obtain an effective size between 1000 and 10,000 as recommended by Hadfield et al.^126,127^ and de Villemereuil^128^, de Villemereuil et al.^129^. The number of sound clips per individual was set to a minimum of 5 and maximum of 50 clips per individual. The first model (G model) determined genetic similarity and residual variances (*n* = 39 individuals; *mean* = 23.3 calls per bird; *sd* = 14.2; *n* = 911 sound clips), and genetic-relatedness was added as a random effect (Fig. 4B.a; Fig. S4). The second model (S model) determined the social and residual variance (*n* = 49 individuals, *mean* = 21.7 calls per bird, *sd* = 13.8, *n* = 1066 sound clips, *min* = 5 and *max* = 50), and social proximity was added as a random effect (Fig. S5). The final model combined genetic, social, and residual variances (G & S model; fixed = trait values ~ 1, random = ~ genetic relatedness + social proximity; *n* = 39 individuals; *mean* = 23.3 calls per bird; *sd* = 14.2; *n* = 911 sound clips, Fig. 4B.b; Fig. S6).

For each model, we plotted the trace of MCMC chains for each acoustic parameter and accessed the curves of traces and posterior density based on their relatively symmetry and unimodality (e.g., Fig. S3). We then reported the estimated percentage and the variances for each model by extracting the post-distribution means, credible intervals, and effective sample sizes for each acoustic parameter (Fig. 4B; Figs. S4–S6). The effective sample sizes ranged from 915.4 to 2883.6 for the G model, 3213.4 and 9,000.0 for S model, and 966.7 and 8,335.8 for the G & S model which satisfied the recommendations of 1000 < effective sample size < 10,000 to run our models^126–129^. Non-overlapping credible intervals indicated a strong separation between variance components (genetic, social, and residual), and a credible interval diverging away from zero best supported the effect of the variance components on call parameters.

Finally, we used DIC for model selection (i.e., based on the smallest DIC values) to determine which model best predicted the proportion of variance components for each acoustic parameter of rifleman feeding calls (Fig. 4A; Table S5). We conducted the above analyses with *R* (v4.2.0; 2022-04-22)^123^.

### Comparisons of rifleman phenotypic vocal variances with a known vocal learner

We compared the phenotypic vocal variance profiles of rifleman to a known vocal learner, the zebra finches to further situate rifleman phenotypic vocal variance along a phenotypic plasticity continuum^45^ and assess whether vocal learning may be a potential mechanism underlying rifleman phenotypic vocal variance (Fig. 4C). Different methods were used to estimate social proximity and acoustic measurements in riflemen and zebra finches, thus we only conducted qualitative rather than quantitative comparisons to assess differences between the phenotypic vocal variance profiles of rifleman and zebra finches. We extracted the phenotypic vocal variance profiles of zebra finches from a reference study by Forstmeier et al.^54^ (Tables A6–A8), which relied on pedigree-based animal models to determine the phenotypic variance and heritability of female zebra finch innate calls and male. The social variance in zebra finches was based on cross-fostering methods (“Foster parent” and “Peer” variances), while for riflemen it was based on geodesic distances between individuals provisioning at the same nest. For zebra finches, we combined the “Foster parent” and “Peer” variances^54^, and we excluded the maternal effects on zebra finches’ vocalizations because maternal effects were not accounted for in the rifleman models. We then selected a subset of variance components of rifleman feeding calls (i.e., four acoustic parameters: duration, entropy, frequency modulation, and mean frequency) and compared them against those of zebra finches (Fig. 4C). We then compared the relative social and genetic ratios between the phenotypic variances of rifleman feeding calls (best explained by our genetic and social proximity model) and non-learned zebra finch female calls, learned male calls, and learned male songs^54^ (Fig. 4C).

### Reporting summary

Further information on research design is available in the Nature Portfolio Reporting Summary linked to this article.

### Supplementary information

Supplementary Information

Reporting Summary

## Data Availability

The data supporting the findings of this study is available in Figshare under a license CC BY 4.0: Moran et al.^130^. *Dataset*. Vocal learning in a vocal non-learner? *Social proximity and vocal convergence shape the calls of the most basal Passeriformes, New Zealand Wrens. 2024. figshare. Dataset*. 10.17608/k6.auckland.25549466.

## Code availability

The R code which performed the study analyses is available in Figshare under a license CC BY 4.0: Moran et al.^130^*. Dataset*. Vocal learning in a vocal non-learner? *Social proximity and vocal convergence shape the calls of the most basal Passeriformes, New Zealand Wrens. 2024. figshare. Dataset*. 10.17608/k6.auckland.25549466.
