# Supplementary material for: Vocal convergence and social proximity shape the calls of the most basal Passeriformes, New Zealand Wrens
Source: Commun Biol. 2024 May 15;7:575. doi: 10.1038/s42003-024-06253-y (PMC11096322; doi:10.1038/s42003-024-06253-y)
Supplement: Supplementary file 1 — Supplementary Information [file 42003_2024_6253_MOESM1_ESM.pdf]

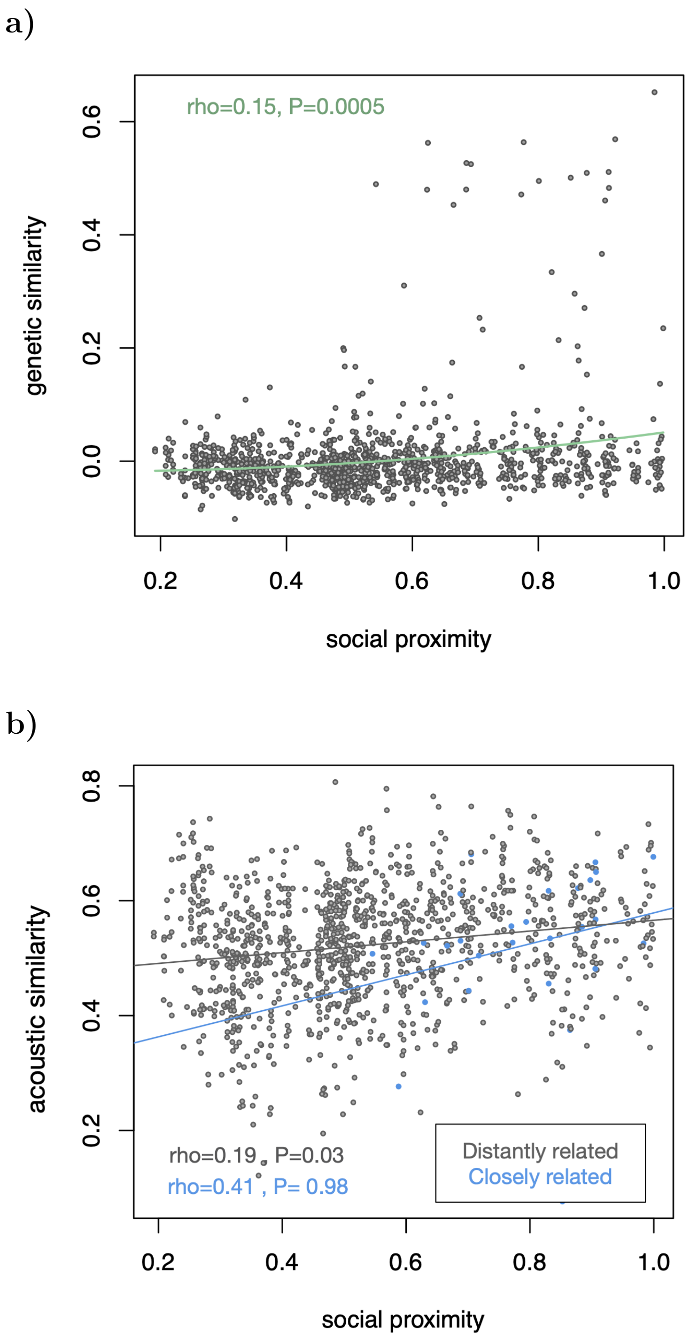

Figure S1: **Relationship between genetic similarity, acoustic similarity and social proximity**. a) Relationship between pairwise genetic similarity and social proximity determined based on relatedness estimates and mean social proximity (based on geodesic distances between nests visited by individuals (1,176 bird pairs across 49 birds). A large social proximity between two birds corresponds to a small geographic distance. Fitted quadratic curve is shown for reference. b) Relationship between pairwise acoustic similarity (mean acoustic cross-correlation) and social proximity either among distantly related pairs of riflemen (1,149 bird pairs across 49 birds, black points) or among closely related pairs of riflemen (27 bird pairs across 29 birds). Fitted linear curves are shown for reference. In both figures, each circle represents one bird pair, and birds without genetic data are not represented.

a)

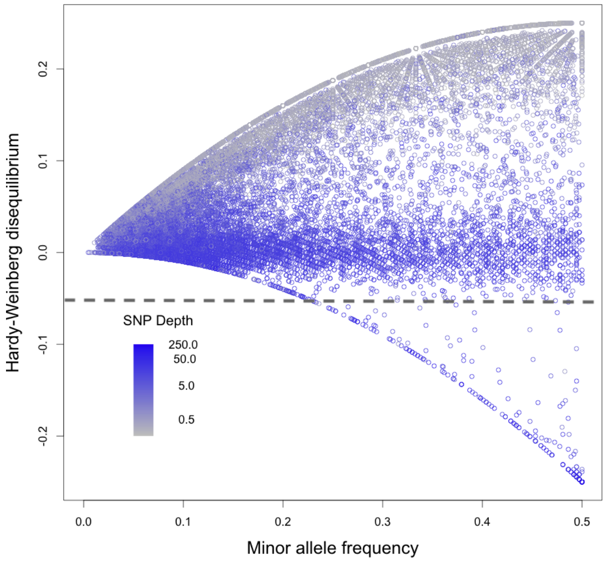

b)

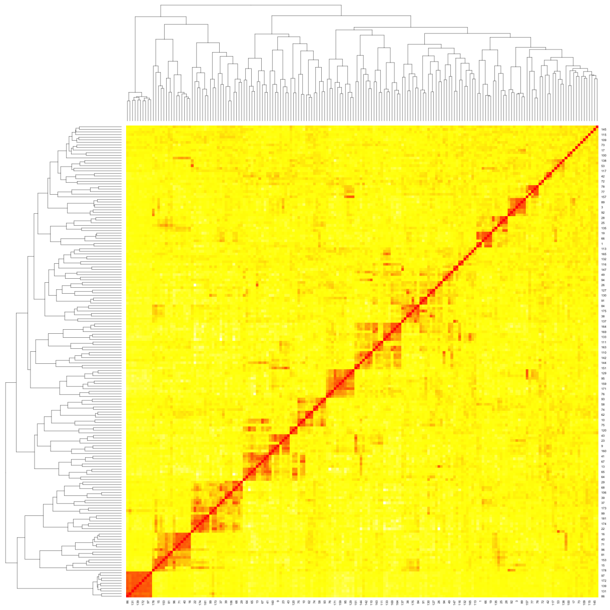

Figure S2: **Hardy-Weinberg disequilibrium finplot, Minor allele frequency and heatmap of the genetic relatedness**. a) Hardy-Weinberg disequilibrium is plotted against Minor Allele Frequency (MAF) showing levels of homozygosity (colored in gray) and heterozygosity (colored in dark purple) with SNP depth in 186 rifleman individuals. The upper edge of the plot has the highest density of low depth SNPs (colored in gray) while the lower edge of the plot has the highest density of high depth SNPs (colored in dark purple) known for their regions of genome duplications and repetitive regions in which putative SNPs do not follow Mendelian inheritance. SNPs with Hardy-Weinberg disequilibrium below -0.05 (dotted line) were removed. b) Heatmap of the genetic relatedness of riflemen. This relatedness heat map is based on the genomic relationship matrix of 186 riflemen created with *KGD* v0.9.5 Kinship using GBS with depth adjustment[67,68]. Individual identity is shown on the vertical and horizontal axis. Colored squares refer to genetic relatedness: the darker red represents genetically closest individuals while the bright yellow represents distantly related individuals.

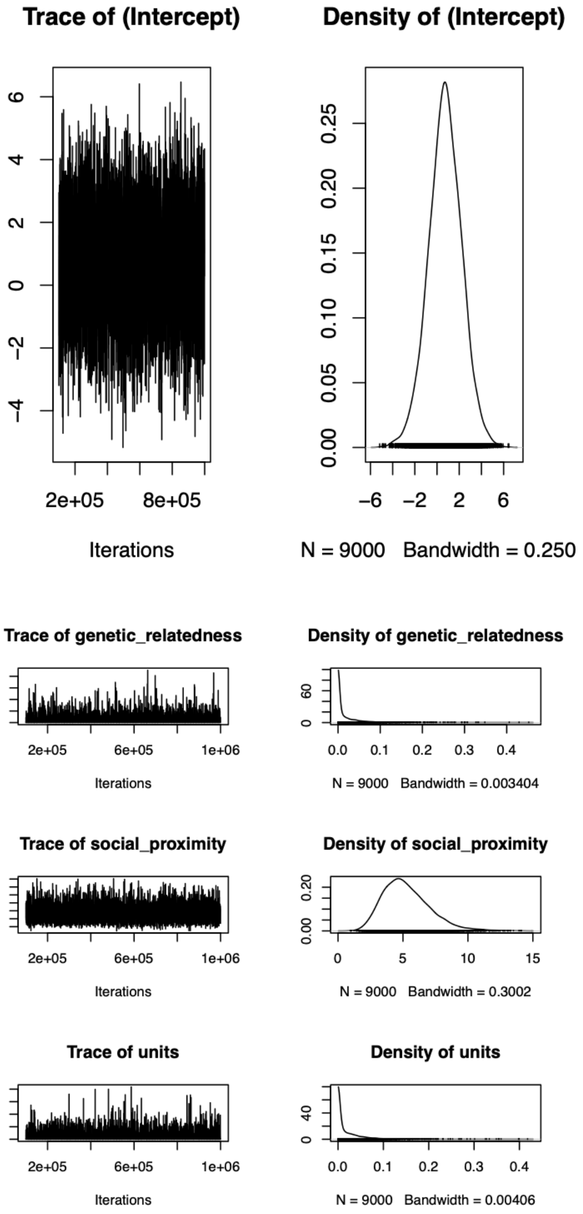

Figure S3: **Selected example of Markov Chain Monte Carlo Trace of the intercept and posterior density for the average slope of the peak frequency contour (Raven_PFC_avgSlope_Hzms) and the variances (six bottom panels)**. Markov Chain Monte Carlo (MCMCglmm) Traces for social and genetic components showing successful convergence. Trace of Intercept are shown on the left panels. The term units refers to the Residual variance.

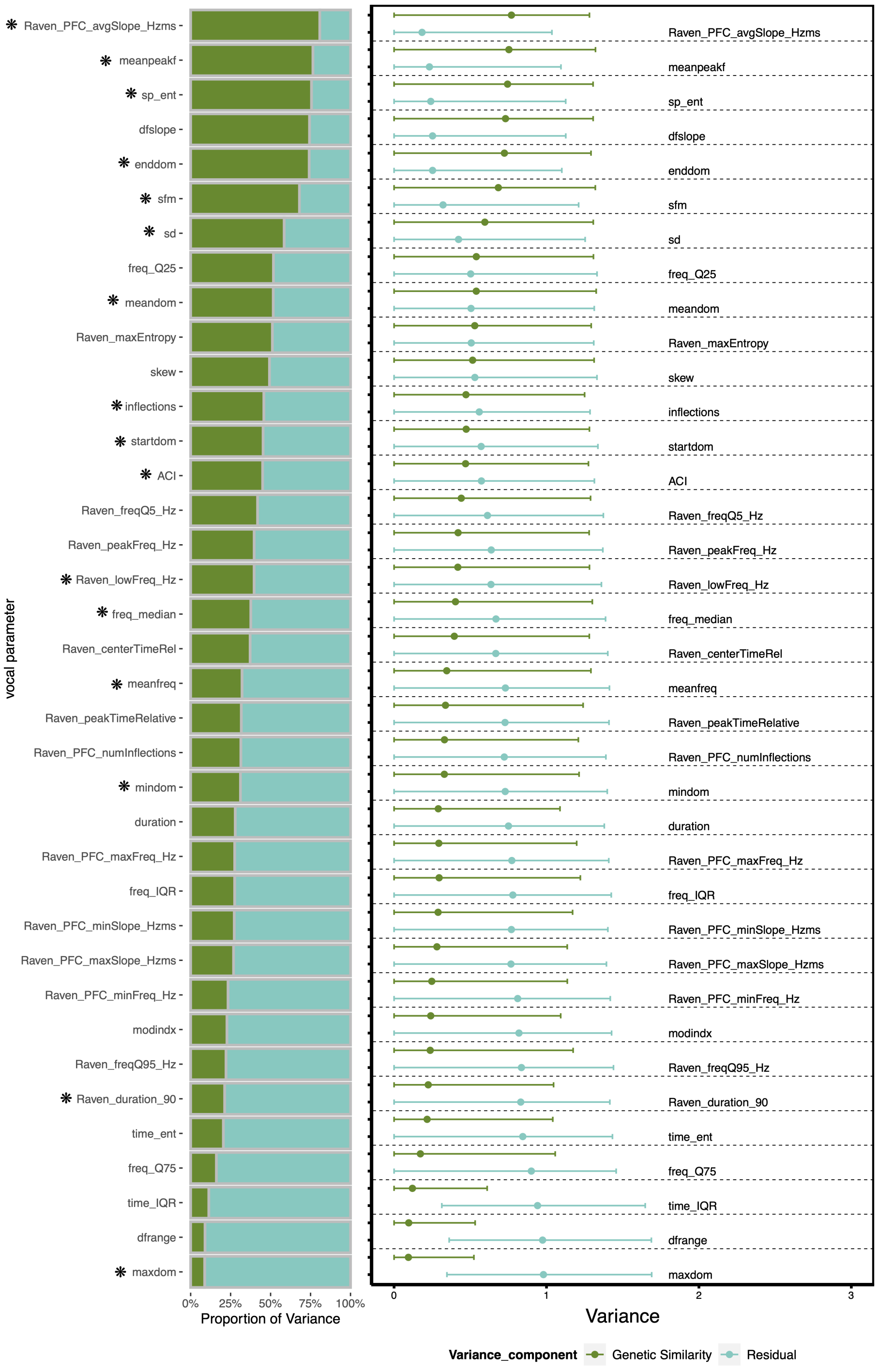

Figure S4: **Variance component estimates of rifleman feeding calls for the genetic similarity model**. Estimation of call variance components followed methods from Thomson *et al.*[41]. This model shows genetic (dark green) and residual (turquoise) variance components with the proportion of variance (in percentage) and the absolute values of the total estimated variance. The variance components of acoustic parameters best explained by this model are marked with a star (* based on Deviance information criterion values; Table S5; n=37 parameters; Table S1; n=38 birds, n=1,067 sound clips). Acoustic parameters were measured using specan from *warbleR* v.1.1.27[65] package.

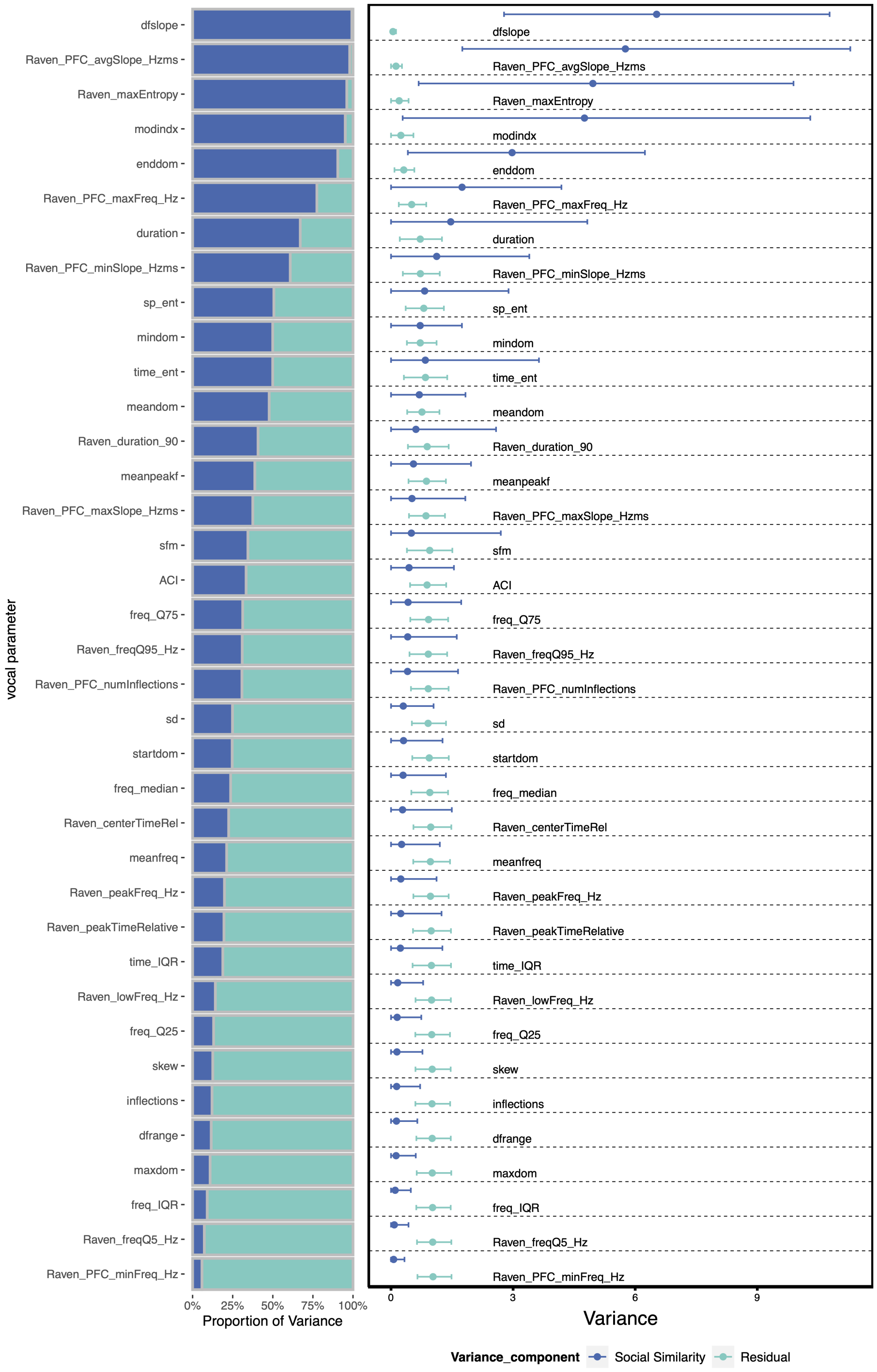

Figure S5: **Variance component estimates of rifleman feeding calls for the social proximity model**. Estimation of call variance for the social proximity model followed methods from Thomson *et al.*[41]. This model shows social (dark blue) and residual (turquoise) variance components with the proportion of variance (in percentage) and the absolute values of the total estimated variance (n=37 parameters; n=47 individuals; Table S1). The social proximity model did not explain any of the variance components of the acoustic parameters (based on Deviance information criterion (DIC) values comparing the three models; Table S5). Acoustic parameters were measured using specan from *warbleR* v.1.1.27[65] package.

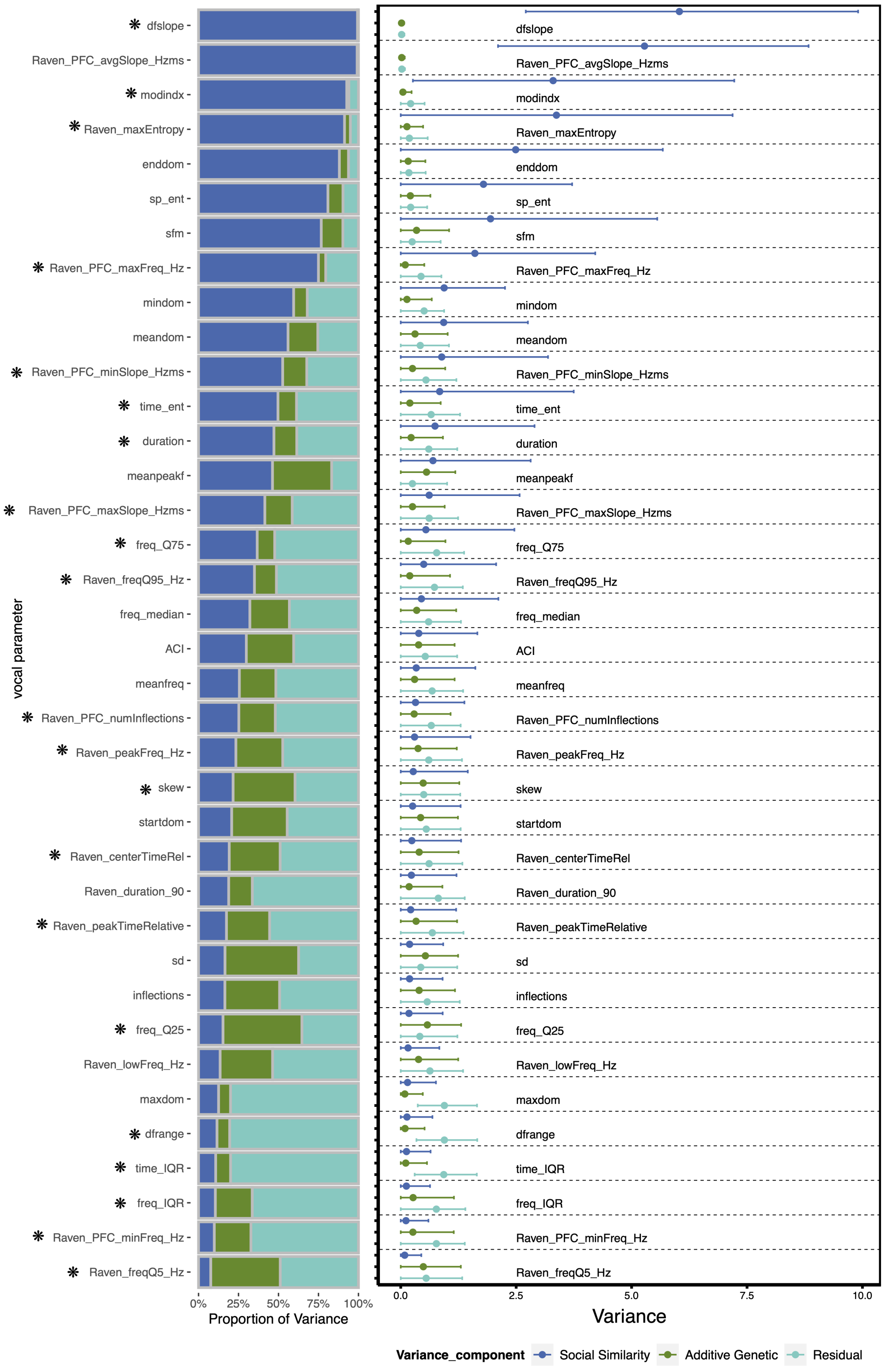

Figure S6: **Variance component estimates of rifleman feeding calls for the social and genetic similarity model**. Estimation of call variance components for the Social and Genetic similarity model followed methods from Thomson *et al.*[41]. This model shows social (dark blue), genetic (dark green) and residual (turquoise) variance components with the proportion of variance (in percentage) and the absolute values of the total estimated variance. The variance components of acoustic parameters best explained by this model are marked with a star (* based on Deviance information criterion (DIC) values comparing three models; Table S5; (n=37 parameters; Table S1; n=38 birds). Rifleman feeding calls were measured using specan from *warbleR* v.1.1.27[65] package.

Supplementary Tables

Table S1: **List of the 37 acoustic parameters used to measure rifleman feeding call features.** Acoustic parameters were measured with WarbleR (specan) v1.1.27[65], Seewave v2.2.0[108] and Raven Pro[107]. All the acoustic parameters are alphabetically ordered. Definitions were extracted and modified from WarbleR (specan)[65] and RavenPro[107].

Vocal parameters	Definition
ACIs	**Acoustic Complexity Index:** Index that measures acoustic complexity of a signal.
dfrange	**Dominant frequency range:** Range of dominant frequency measured across the acoustic signal.
dfslope	**Dominant frequency slope:** the change in the slope of the dominant frequency through time((enddom-startdom)/duration). Units: kHz/s.
duration	**Duration:** Length of signal. Units: s.
enddom	**End dominant frequency:** Dominant frequency measurement at the end of the signal.
freq.IQR	**Interquartile frequency range:** Frequency range between 'freq.Q25' and 'freq.Q75'. Units: kHz.
freq.median	**Median frequency:** The frequency at which the signal is divided in two frequency intervals of equal energy. Units: kHz.
freq.Q25	**First quartile frequency:** The frequency at which the signal is divided in two frequency intervals of 25(%)and 75 (%) energy respectively. Units: kHz.
freq.Q75	**Third quartile frequency:** The frequency at which the signal is divided in two frequency intervals of 75 (%) and 25 (%) energy respectively. Units: kHz.
inflections	**Inflections:** The number of inflections in a frequency contour (or any time series).
maxdom	**Maximum dominant frequency:** Maximum of dominant frequency measured across the acoustic signal.
meandom	**Mean dominant frequency:** Mean dominant frequency measured across the acoustic signal.
meanfreq	**Mean frequency :** Mean of frequency spectrum (i.e., weighted average of frequency by amplitude within supplied band pass). Units: kHz.
meanpeakf	**Mean peak frequency:** Frequency with highest energy from the mean frequency spectrum (see meanspec). Typically more consistent than peakf.
mindom	**Minimum dominant frequency:** Minimum of dominant frequency measured across the acoustic signal.
modindx	**Modulation index:** Calculated as the cumulative absolute difference between adjacent measurements of dominant frequencies divided by the dominant frequency range. 1 means the signal is not modulated.
sd	**Standard deviation:** Standard deviation of frequency. Units: kHz.
sfm	**Spectral flatness:** Similar to sp.ent (Pure tone $\sim$ 0; noisy $\sim$ 1).
skew	**Skewness:** Asymmetry of the spectrum (see note in specprop description).
sp.ent	**Spectral entropy:** Energy distribution of the frequency spectrum. Pure tone $\sim$ 0; noisy $\sim$ 1.
startdom	**Start of dominant frequency:** Dominant frequency measurement at the start of the signal
time.ent	**Time entropy:** Energy distribution on the time envelope. Pure tone $\sim$ 0; noisy $\sim$ 1.
time.IQR	**Interquartile time range:** Time range between 'time.Q25' and 'time.Q75'. Units: s.
RavenPro center Time Rel	**RavenPro centre Time Relative.**
RavenPro duration 90	**RavenPro duration:** The difference between the 5% and 95% times. Units: s
RavenPro freq Q5 Hz	**RavenPro Frequency 5% quartile:** The frequency that divides the selection into two frequency intervals containing 5% and 95% of the energy in the selection. The computation of this measurement is similar to that of centre Frequency, except that the summed energy has to exceed 5% of the total energy instead of 50%. Units: Hz.
RavenPro freq Q95 Hz	**RavenPro Frequency 95%:** The frequency that divides the selection into two frequency intervals containing 95% and 5% of the energy in the selection. The computation of this measurement is similar to that of centre Frequency, except that the summed energy has to exceed 95% of the total energy instead of 50%.
RavenPro low Freq Hz	**RavenPro Low Frequency:** The lower frequency bound of the selection. Units: Hz.
RavenPro maxEntropy	**RavenPro Maximum Entropy:** The maximum entropy calculated for a spectrogram slice within the selection bounds.
RavenPro peak Freq Hz	**RavenPro Peak Frequency:** The frequency at which Max Power/ Peak Power occurs within the selection. If Max Power/ Peak Power occurs at more than one time and/or frequency, the lowest frequency at Max Time at which Max Power/ Peak Power occurs. Units: Hz.
RavenPro peak Time Relative	**RavenPro Peak Time Relative:** In a waveform view, the first time in a selection at which a sample with amplitude equal to Peak Amplitude occurs, the time expressed as a proportion of the duration of the selection. In a spectrogram view, the first time in a selection at which a spectrogram bin with power density equal to Peak Power occurs, the time expressed as a proportion of the duration of the selection.
RavenPro PFC avgSlope Hz ms	**Peak Frequency Contour Average Slope:** Mean of the Peak Frequency Contour Slope.
RavenPro PFC maxFreq Hz	**Peak Frequency Contour Max Frequency:** Maximum of the Peak Frequency Contour.
RavenPro PFC maxSlope Hzms	**Peak Frequency Contour Max Slope:** Maximum of the Peak Frequency Contour Slope.
RavenPro PFC minFreq Hz	**Peak Frequency Contour Min Frequency:** Minimum of the Peak Frequency Contour.
RavenPro PFC minSlope Hzms	**Peak Frequency Contour Min Slope:** Minimum of the Peak Frequency Contour Slope Series.
RavenPro PFC num Inflections	**Peak Frequency Contour Number of Inflection Points:** Number of times the slope changes sign in Peak Frequency Contour Slope.

Table S2: **Spearman's correlation between the genetic distance and mean absolute difference of each acoustic parameter.** Spearman's correlations (Mantel ϱ; two-sided) and significance values are presented for each acoustic parameter (n=1,110 call clips; n=49 birds; *npairs*=1,176). None of the acoustic parameters (n=37 acoustic parameters) were significantly correlated with genetic proximity.

**Acoustic parameter**	**Mantel ϱ**	**Significance**
ACI	0.048	0.22
dfrange	-0.059	0.15
dfslope	-0.035	0.44
duration	0.012	0.76
enddom	0.039	0.39
freq_IQR	0.017	0.68
freq_median	0.002	0.96
freq_Q25	-0.014	0.73
freq_Q75	0.004	0.92
inflections	0.043	0.31
maxdom	-0.080	0.07
meandom	-0.013	0.75
meanfreq	-0.023	0.56
meanpeakf	0.001	0.99
mindom	-0.009	0.82
modindx	0.084	0.06
sd	-0.027	0.51
sfm	0.027	0.48
skew	-0.016	0.72
sp_ent	0.019	0.65
startdom	-0.011	0.81
time_ent	0.011	0.76
time_IQR	-0.010	0.80
Raven_centerTimeRel	-0.025	0.55
Raven_duration_90	-0.001	0.98
Raven_freqQ5_Hz	-0.014	0.73
Raven_freqQ95_Hz	-0.031	0.48
Raven_lowFreq_Hz	0.032	0.40
Raven_maxEntropy	0.009	0.83
Raven_peakFreq_Hz	-0.013	0.78
Raven_peakTimeRelative	0.016	0.74
Raven_PFC_avgSlope_Hzms	0.001	0.98
Raven_PFC_maxFreq_Hz	0.022	0.60
Raven_PFC_maxSlope_Hzms	-0.024	0.61
Raven_PFC_minFreq_Hz	0.007	0.85
Raven_PFC_minSlope_Hzms	0.037	0.41
Raven_PFC_numInflections	-0.013	0.76

Table S3: **Spearman's correlation between the social proximity and mean absolute difference of 37 acoustic parameters in distantly related pairs of birds**. Spearman's correlations (Mantel ϱ; two-sided) and significance values are presented for distantly pairs of related riflemen (1,149 pairs of birds across 49 birds). Significant correlations between acoustic parameters and social proximity are highlighted.

**Acoustic parameters**	**Distantly related**	
	Mantel ϱ	Significance
ACI	-0.09	0.17
dfrange	0.07	0.35
dfslope	-0.27	0.003
duration	0.15	0.04
enddom	-0.14	0.13
freq_IQR	0.06	0.49
freq_median	-0.004	0.96
freq_Q25	0.03	0.73
freq_Q75	-0.02	0.76
inflections	0.20	0.01
maxdom	0.03	0.69
meandom	0.05	0.52
meanfreq	-0.02	0.76
meanpeakf	0.05	0.59
mindom	0.03	0.64
modindx	0.12	0.18
sd	-0.09	0.26
sfm	-0.06	0.40
skew	-0.02	0.85
sp_ent	-0.16	0.05
startdom	0.13	0.20
time_ent	0.07	0.21
time_IQR	0.03	0.70
Raven_centerTimeRel	-0.10	0.19
Raven_duration_90	0.06	0.33
Raven_freqQ5_Hz	0.03	0.72
Raven_freqQ95_Hz	-0.08	0.33
Raven_lowFreq_Hz	-0.08	0.24
Raven_maxEntropy	-0.17	0.02
Raven_peakFreq_Hz	-0.03	0.70
Raven_peakTimeRelative	-0.12	0.21
Raven_PFC_avgSlope_Hzms	-0.20	0.03
Raven_PFC_maxFreq_Hz	-0.16	0.06
Raven_PFC_maxSlope_Hzms	-0.16	0.10
Raven_PFC_minFreq_Hz	-0.03	0.71
Raven_PFC_minSlope_Hzms	-0.22	0.01
Raven_PFC_numInflections	0.05	0.54

Table S4: **Mutual pairwise spearman correlations among the 7 vocal parameters which had significant correlations with social proximity (Table S3).**

**vocal__param1**	**vocal__param2**	**correlation**
inflections	duration	0.74
Raven__PFC__avgSlope__Hzms	dfslope	0.46
Raven__maxEntropy	inflections	0.45
inflections	sp__ent	0.44
Raven__maxEntropy	sp__ent	0.41
Raven__maxEntropy	duration	0.41
sp__ent	duration	0.33
Raven__PFC__minSlope__Hzms	Raven__PFC__avgSlope__Hzms	0.30
Raven__PFC__minSlope__Hzms	dfslope	0.05
Raven__PFC__minSlope__Hzms	Raven__maxEntropy	-0.03
Raven__PFC__minSlope__Hzms	sp__ent	-0.16
Raven__PFC__minSlope__Hzms	duration	-0.21
Raven__PFC__minSlope__Hzms	inflections	-0.22
dfslope	sp__ent	-0.24
Raven__PFC__avgSlope__Hzms	Raven__maxEntropy	-0.25
Raven__PFC__avgSlope__Hzms	sp__ent	-0.26
Raven__PFC__avgSlope__Hzms	inflections	-0.30
inflections	dfslope	-0.32
Raven__maxEntropy	dfslope	-0.35
Raven__PFC__avgSlope__Hzms	duration	-0.50
dfslope	duration	-0.50

Table S5: **Deviance Information criterion (DIC) for all three of the multiple-matrix animal models and for each acoustic parameter (n=37)**. The multiple-matrix animal models include the genetic similarity model (G model), the social proximity model (S model), and the genetic similarity and social proximity model (G & S model). For each acoustic parameter, models with the smallest DIC are highlighted in yellow. Models with smaller DIC best explain the phenotypic call variance components of the acoustic parameter.

**Acoustic parameter**	**G model**	**S model**	**G & S model**
ACI	6.7	138.1	7.4
dfrange	107.8	141.9	107.2
dfslope	-114.9	-42.6	-131.5
duration	74.5	129.1	52.4
enddom	-109.7	96.7	-50.7
freq_IQR	52.2	142.0	61.0
freq_median	11.3	140.6	14.1
freq_Q25	-43.0	141.7	-63.0
freq_Q75	85.1	139.6	76.7
inflections	11.3	141.9	28.0
maxdom	105.0	141.9	105.5
meandom	-32.5	133.1	-9.9
meanfreq	27.9	140.8	29.9
meanpeakf	-129.3	137.7	-105.3
mindom	46.8	130.8	67.6
modindx	78.0	72.9	27.0
sd	-42.2	139.2	-36.6
sfm	-96.4	139.0	-61.0
skew	-26.7	141.9	-28.5
sp_ent	-121.6	134.7	-37.2
startdom	-3.1	140.0	-1.6
time_ent	84.4	135.6	57.9
time_IQR	105.7	141.4	104.9
Raven_centerTimeRel	15.3	141.0	7.4
Raven_duration_90	85.0	138.0	90.4
Raven_freqQ5_Hz	-5.2	142.2	-24.6
Raven_freqQ95_Hz	64.9	139.4	62.3
Raven_lowFreq_Hz	15.5	141.6	19.5
Raven_maxEntropy	-26.5	70.6	-39.9
Raven_peakFreq_Hz	12.7	141.1	12.5
Raven_peakTimeRelative	34.4	141.3	29.2
Raven_PFC_avgSlope_Hzms	-145.3	29.5	-117.5
Raven_PFC_maxFreq_Hz	58.8	116.9	55.4
Raven_PFC_maxSlope_Hzms	70.7	137.3	44.9
Raven_PFC_minFreq_Hz	74.0	142.3	62.6
Raven_PFC_minSlope_Hzms	57.8	130.9	21.2
Raven_PFC_numInflections	52.1	139.4	50.7
