# Supplementary material for: Vocal convergence and social proximity shape the calls of the most basal Passeriformes, New Zealand Wrens
Source: Commun Biol. 2024 May 15;7:575. doi: 10.1038/s42003-024-06253-y (PMC11096322; doi:10.1038/s42003-024-06253-y)
Supplement: Supplementary file 2 — Reporting Summary [file 42003_2024_6253_MOESM2_ESM.pdf]

nature portfolio

Corresponding author(s): COMMSBIO-22-3013D

Last updated by author(s): April 6th, 2024

Reporting Summary

Nature Portfolio wishes to improve the reproducibility of the work that we publish. This form provides structure for consistency and transparency in reporting. For further information on Nature Portfolio policies, see our Editorial Policies and the Editorial Policy Checklist.

Statistics

For all statistical analyses, confirm that the following items are present in the figure legend, table legend, main text, or Methods section.

n/a	Confirmed	
☐	☒	The exact sample size (*n*) for each experimental group/condition, given as a discrete number and unit of measurement
☐	☒	A statement on whether measurements were taken from distinct samples or whether the same sample was measured repeatedly
☐	☒	The statistical test(s) used AND whether they are one- or two-sided<br>*Only common tests should be described solely by name; describe more complex techniques in the Methods section.*
☐	☒	A description of all covariates tested
☐	☒	A description of any assumptions or corrections, such as tests of normality and adjustment for multiple comparisons
☐	☒	A full description of the statistical parameters including central tendency (e.g. means) or other basic estimates (e.g. regression coefficient) AND variation (e.g. standard deviation) or associated estimates of uncertainty (e.g. confidence intervals)
☐	☒	For null hypothesis testing, the test statistic (e.g. F, t, r) with confidence intervals, effect sizes, degrees of freedom and P value noted<br>*Give P values as exact values whenever suitable.*
☐	☒	For Bayesian analysis, information on the choice of priors and Markov chain Monte Carlo settings
☐	☒	For hierarchical and complex designs, identification of the appropriate level for tests and full reporting of outcomes
☐	☒	Estimates of effect sizes (e.g. Cohen's d, Pearson's r), indicating how they were calculated

Our web collection on statistics for biologists contains articles on many of the points above.

Software and code

Policy information about availability of computer code

Data collection: No software or code were used to collect data

Data analysis: Custom codes were used for the data analysis of this study. All data analysis and data input are available at: 1) 10.17608/k6.auckland.25549466 ; 2) https://github.com/UoA-eResearch/bird_recognition; and 3) https://uoa-eresearch.github.io/bird_recognition/time_sync_check.nb.html

#Licence CCBY 4.0
You can share, copy, and modify this dataset so long as you give appropriate credit (see below), provide a link to the CC BY license, and indicate if changes were made, but you may not do so in a way that suggests the rights holder has endorsed you or your use of the dataset. Note that further permission may be required for any content within the dataset that is identified as belonging to a third party.
If the datasets and R scripts are shared, copied, and modified, please cite:
Moran et al. 2024. "Social proximity and vocal convergence shape the calls of the most basal Passeriformes, New Zealand Wrens"

For manuscripts utilizing custom algorithms or software that are central to the research but not yet described in published literature, software must be made available to editors and reviewers. We strongly encourage code deposition in a community repository (e.g. GitHub). See the Nature Portfolio guidelines for submitting code & software for further information.

Data

Policy information about availability of data

All manuscripts must include a data availability statement. This statement should provide the following information, where applicable:

- Accession codes, unique identifiers, or web links for publicly available datasets
- A description of any restrictions on data availability
- For clinical datasets or third party data, please ensure that the statement adheres to our policy

All data analysis and data input are available at: 1) 10.17608/k6.auckland.25549466 ; 2) https://github.com/UoA-eResearch/bird_recognition; and 3) https://uoa-eresearch.github.io/bird_recognition/time_sync_check.nb.html

#Licence CCBY 4.0
You can share, copy, and modify this dataset so long as you give appropriate credit (see below), provide a link to the CC BY license, and indicate if changes were made, but you may not do so in a way that suggests the rights holder has endorsed you or your use of the dataset. Note that further permission may be required for any content within the dataset that is identified as belonging to a third party.
If the datasets and R scripts are shared, copied, and modified, please cite:
Moran et al. 2024. "Social proximity and vocal convergence shape the calls of the most basal Passeriformes, New Zealand Wrens"

Human research participants

Policy information about studies involving human research participants and Sex and Gender in Research.

Reporting on sex and gender	*Use the terms sex (biological attribute) and gender (shaped by social and cultural circumstances) carefully in order to avoid confusing both terms. Indicate if findings apply to only one sex or gender; describe whether sex and gender were considered in study design whether sex and/or gender was determined based on self-reporting or assigned and methods used. Provide in the source data disaggregated sex and gender data where this information has been collected, and consent has been obtained for sharing of individual-level data; provide overall numbers in this Reporting Summary. Please state if this information has not been collected. Report sex- and gender-based analyses where performed, justify reasons for lack of sex- and gender-based analysis.*
Population characteristics	*Describe the covariate-relevant population characteristics of the human research participants (e.g. age, genotypic information, past and current diagnosis and treatment categories). If you filled out the behavioural & social sciences study design questions and have nothing to add here, write "See above."*
Recruitment	*Describe how participants were recruited. Outline any potential self-selection bias or other biases that may be present and how these are likely to impact results.*
Ethics oversight	*Identify the organization(s) that approved the study protocol.*

Note that full information on the approval of the study protocol must also be provided in the manuscript.

Field-specific reporting

Please select the one below that is the best fit for your research. If you are not sure, read the appropriate sections before making your selection.

☐ Life sciences ☐ Behavioural & social sciences ☒ Ecological, evolutionary & environmental sciences

For a reference copy of the document with all sections, see nature.com/documents/nr-reporting-summary-flat.pdf

Ecological, evolutionary & environmental sciences study design

All studies must disclose on these points even when the disclosure is negative.

Study description	This study uses a combination of acoustic, spatial, and genetic data and methods to investigate the potential presence of vocal learning abilities in the rifleman (Acanthisitta chloris).
Research sample	We collected acoustic, genetic and social samples from rifleman (Acanthisitta chloris) from Boundary Stream mainland Island reserve, New Zealand. We collected feeding zip calls, blood samples (i.e., to determine genetic relatedness), and geolocations of individuals at the nests (i.e., as a proxy for social proximity). Rationale for sample choice: Riflemen are assumed to be vocal non-learners, but the vocal learning abilities of the rifleman have never been tested. We investigated the potential presence of vocal learning by comparing the vocal phenotype of rifleman from difference genetic and social backgrounds. Details about samples: adults, females and males. We also used a preexisting dataset from Forstmeier et al.2009 to compare the quantitative traits from zebra finches (i.e., vocal learners) with those of the rifleman.
Sampling strategy	The sample size was limited by natural constraints in the field, such as the number of nests found and the number of individuals caught during banding sessions. The number of feeding call recordings per individual depended on the number of visits at nest from

	focal individuals, and also depended on the types of methods used to record these calls. All recorded feeding calls from the RFID were used for the vocal signature analysis, while a subset of feeding calls (<50 calls per individuals) from all recording methods combine together, were used for the remaining of the analysis. This latter strategy contributed to a more balanced dataset (i.e., by reducing the number of calls per individual from thousands to <50 calls). The number 50 was an arbitrary number which capture the within-variation of individuals' feeding calls (i.e., based on the vocal signature analysis).
Data collection	Data collection procedure: Feeding calls for each focal individual were collected using a combination of methods (focal recordings, RFID, ARU, and trail cameras). The GPS location of each focal individual were collected using MapsMe. Blood samples for DNA extraction were collected during banding sessions using brachial venipuncture with a BD PrecisionGlide sterile needle (26G 1/2-0.45mm* 13mm) and a 70 uL capillary tude (Microhaematocrit tubes) and were preserved in 95% ethanol which were then temporarily stored at 4 degree Celsius in the field. The acoustic and geolocation data was collected by Ines G. Moran with additional help from co-authors YY. Loo, S. Withers, S. Louca, K. Cain and other helpers T. and J. Wells, J. Leung, A. Menzies, M. Bidmead, F. Patterson. The genetic data was collected by Ines Moran with additional help from co-authors YY. Loo, S. Withers, K. Cain, and other helpers J. Leung, A. Menzies, and M. Bidmead.
Timing and spatial scale	Field data collection lasted six months per year during two austral breeding seasons from September to February between 2018-2020 at the Boundary Stream mainland Island reserve, New Zealand (39°06'15.8"S, 176°48'06.1"E).
Data exclusions	*If no data were excluded from the analyses, state so OR if data were excluded, describe the exclusions and the rationale behind them, indicating whether exclusion criteria were pre-established.*
Reproducibility	We used R scripts and established pipelines to facilitate the reproducibility of our analyses.
Randomization	*Describe how samples/organisms/participants were allocated into groups. If allocation was not random, describe how covariates were controlled. If this is not relevant to your study, explain why.*
Blinding	Blinding was not relevant to the study. Data collected was gathered as nests were discovered so no potential biases occurred.

Did the study involve field work? ☒ Yes ☐ No

Field work, collection and transport

Field conditions	We monitored the rifleman populations of Boundary Stream Mainland Island daily over a period of 6 months each year from late austral winter to summer. Field conditions varied depending on the seasons.
Location	Boundary Stream Mainland Island research, New Zealand (39°06'15.8"S, 176°48'06.1"E), and Mohi Bush (39°51'25.34"S, 176° 54'7.49"E). Rifleman population at Mohi Mush is a geographically close but genetically distant population to the population from Boundary Stream Mainland Island reserve. Genetic samples from Mohi Bush enabled use to increase our genetic sample size to 186 individuals, which in turn provided us with a more robust genetic relatedness estimate.
Access & import/export	This research was approved and facilitated by the mana whenua from the Maungaharuru region and the Department of Conservation, New Zealand (Department of Conservation permit obtained in 2018, act No FAU 55391). This research was approved by the University if Auckland Animal Ethics Committee (Approval no. 001866). Bird capture and banding activities were conducted under the New Zealand National Bird Banding Scheme.
Disturbance	Bird capture and banding activities were conducted with care following the New Zealand National Bird Banding Scheme. Activities around wild nests (i.e. monitoring observations) followed good field practices which respected wildlife (e.g. reduction of time spent close to nests)

Reporting for specific materials, systems and methods

We require information from authors about some types of materials, experimental systems and methods used in many studies. Here, indicate whether each material, system or method listed is relevant to your study. If you are not sure if a list item applies to your research, read the appropriate section before selecting a response.

Materials & experimental systems

n/a	Involved in the study	
☐	☐	Antibodies
☐	☐	Eukaryotic cell lines
☐	☐	Palaeontology and archaeology
☐	☒	Animals and other organisms
☐	☐	Clinical data
☐	☐	Dual use research of concern

Methods

n/a	Involved in the study	
☐	☐	ChIP-seq
☐	☐	Flow cytometry
☐	☐	MRI-based neuroimaging

Antibodies

Antibodies used	*Describe all antibodies used in the study; as applicable, provide supplier name, catalog number, clone name, and lot number.*
Validation	*Describe the validation of each primary antibody for the species and application, noting any validation statements on the manufacturer's website, relevant citations, antibody profiles in online databases, or data provided in the manuscript.*

Eukaryotic cell lines

Policy information about cell lines and Sex and Gender in Research

Cell line source(s)	*State the source of each cell line used and the sex of all primary cell lines and cells derived from human participants or vertebrate models.*
Authentication	*Describe the authentication procedures for each cell line used OR declare that none of the cell lines used were authenticated.*
Mycoplasma contamination	*Confirm that all cell lines tested negative for mycoplasma contamination OR describe the results of the testing for mycoplasma contamination OR declare that the cell lines were not tested for mycoplasma contamination.*
Commonly misidentified lines (See ICLAC register)	*Name any commonly misidentified cell lines used in the study and provide a rationale for their use.*

Palaeontology and Archaeology

Specimen provenance	*Provide provenance information for specimens and describe permits that were obtained for the work (including the name of the issuing authority, the date of issue, and any identifying information). Permits should encompass collection and, where applicable, export.*
Specimen deposition	*Indicate where the specimens have been deposited to permit free access by other researchers.*
Dating methods	*If new dates are provided, describe how they were obtained (e.g. collection, storage, sample pretreatment and measurement), where they were obtained (i.e. lab name), the calibration program and the protocol for quality assurance OR state that no new dates are provided.*

☐ Tick this box to confirm that the raw and calibrated dates are available in the paper or in Supplementary Information.

Ethics oversight	*Identify the organization(s) that approved or provided guidance on the study protocol, OR state that no ethical approval or guidance was required and explain why not.*

Note that full information on the approval of the study protocol must also be provided in the manuscript.

Animals and other research organisms

Policy information about studies involving animals; ARRIVE guidelines recommended for reporting animal research, and Sex and Gender in Research

Laboratory animals	This study did not involve laboratory animals
Wild animals	For monitoring and identification purposes, we caught individuals using mist-nets and speakers with conspecific lure calls. We then sexed and aged adults and fledglings based on their sexual dimorphism in size and coloration. Then, we banded individuals with uique color band combinations. One of the leg bands was equipped with a Passive Integrated Transponder (2.3mm EM4102 PIT Tag; Eccel Technology Embedded RFID) that could be read by Radio-frequency identification receivers (RFID; engineered by the University of Auckland) placed at nests. Once sexed, aged and banded (i.e., and activity that takes a few minutes), birds were released back to the location where they were caught.
Reporting on sex	Sexing was based on plumage dimorphism. Based on unpublished results, no acoustic differences exist between female and male feeding zip calls; consequently we did not split our results by sex.
Field-collected samples	Blood samples collected from 2018-2020 were preserved in 95% ethanol and temporarily stored at 4 degree Celsius in the field before being transferred for long-term storage at -20 degree Celcius. In 2020, following the end of the 2019-2020 field season, individual blood samples were extracted using Qiagen DNeasy Blood and Tissue kit. Once extracted, DNA aliquots were then dried (3hrs at 30 degree Celcius) and sealed for shipping to AgResearch, Mosgiel, New Zealand, where a Genotyping-By-Sequencing (GBS) protocol was carried out.
Ethics oversight	The Department of Conservation permit was obtained in 2018, act No FAU 55391. This research was approved by the University of Auckland Animal Ethics Commitee (Approval no. 001866). Bird capture and banding activities were conducted under the New Zealand National Bird Banding Scheme.

Note that full information on the approval of the study protocol must also be provided in the manuscript.

Clinical data

Policy information about clinical studies

All manuscripts should comply with the ICMJE guidelines for publication of clinical research and a completed CONSORT checklist must be included with all submissions.

Clinical trial registration	*Provide the trial registration number from ClinicalTrials.gov or an equivalent agency.*
Study protocol	*Note where the full trial protocol can be accessed OR if not available, explain why.*
Data collection	*Describe the settings and locales of data collection, noting the time periods of recruitment and data collection.*
Outcomes	*Describe how you pre-defined primary and secondary outcome measures and how you assessed these measures.*

Dual use research of concern

Policy information about dual use research of concern

Hazards

Could the accidental, deliberate or reckless misuse of agents or technologies generated in the work, or the application of information presented in the manuscript, pose a threat to:

No	Yes	
☒	☐	Public health
☒	☐	National security
☒	☐	Crops and/or livestock
☒	☐	Ecosystems
☒	☐	Any other significant area

Experiments of concern

Does the work involve any of these experiments of concern:

No	Yes	
☒	☐	Demonstrate how to render a vaccine ineffective
☒	☐	Confer resistance to therapeutically useful antibiotics or antiviral agents
☒	☐	Enhance the virulence of a pathogen or render a nonpathogen virulent
☒	☐	Increase transmissibility of a pathogen
☒	☐	Alter the host range of a pathogen
☒	☐	Enable evasion of diagnostic/detection modalities
☒	☐	Enable the weaponization of a biological agent or toxin
☒	☐	Any other potentially harmful combination of experiments and agents

ChIP-seq

Data deposition

☐ Confirm that both raw and final processed data have been deposited in a public database such as GEO.

☐ Confirm that you have deposited or provided access to graph files (e.g. BED files) for the called peaks.

Data access links<br>*May remain private before publication.*	*For "Initial submission" or "Revised version" documents, provide reviewer access links. For your "Final submission" document, provide a link to the deposited data.*
Files in database submission	*Provide a list of all files available in the database submission.*
Genome browser session<br>(e.g. UCSC)	*Provide a link to an anonymized genome browser session for "Initial submission" and "Revised version" documents only, to enable peer review. Write "no longer applicable" for "Final submission" documents.*

Methodology

Replicates	*Describe the experimental replicates, specifying number, type and replicate agreement.*
Sequencing depth	*Describe the sequencing depth for each experiment, providing the total number of reads, uniquely mapped reads, length of reads and*

Sequencing depth | *whether they were paired- or single-end.*

Antibodies | *Describe the antibodies used for the ChIP-seq experiments; as applicable, provide supplier name, catalog number, clone name, and lot number.*

Peak calling parameters | *Specify the command line program and parameters used for read mapping and peak calling, including the ChIP, control and index files used.*

Data quality | *Describe the methods used to ensure data quality in full detail, including how many peaks are at FDR 5% and above 5-fold enrichment.*

Software | *Describe the software used to collect and analyze the ChIP-seq data. For custom code that has been deposited into a community repository, provide accession details.*

Flow Cytometry

Plots

Confirm that:

- [ ] The axis labels state the marker and fluorochrome used (e.g. CD4-FITC).
- [ ] The axis scales are clearly visible. Include numbers along axes only for bottom left plot of group (a 'group' is an analysis of identical markers).
- [ ] All plots are contour plots with outliers or pseudocolor plots.
- [ ] A numerical value for number of cells or percentage (with statistics) is provided.

Methodology

Sample preparation | *Describe the sample preparation, detailing the biological source of the cells and any tissue processing steps used.*

Instrument | *Identify the instrument used for data collection, specifying make and model number.*

Software | *Describe the software used to collect and analyze the flow cytometry data. For custom code that has been deposited into a community repository, provide accession details.*

Cell population abundance | *Describe the abundance of the relevant cell populations within post-sort fractions, providing details on the purity of the samples and how it was determined.*

Gating strategy | *Describe the gating strategy used for all relevant experiments, specifying the preliminary FSC/SSC gates of the starting cell population, indicating where boundaries between "positive" and "negative" staining cell populations are defined.*

- [ ] Tick this box to confirm that a figure exemplifying the gating strategy is provided in the Supplementary Information.

Magnetic resonance imaging

Experimental design

Design type | *Indicate task or resting state; event-related or block design.*

Design specifications | *Specify the number of blocks, trials or experimental units per session and/or subject, and specify the length of each trial or block (if trials are blocked) and interval between trials.*

Behavioral performance measures | *State number and/or type of variables recorded (e.g. correct button press, response time) and what statistics were used to establish that the subjects were performing the task as expected (e.g. mean, range, and/or standard deviation across subjects).*

Acquisition

Imaging type(s) | *Specify: functional, structural, diffusion, perfusion.*

Field strength | *Specify in Tesla*

Sequence & imaging parameters | *Specify the pulse sequence type (gradient echo, spin echo, etc.), imaging type (EPI, spiral, etc.), field of view, matrix size, slice thickness, orientation and TE/TR/flip angle.*

Area of acquisition | *State whether a whole brain scan was used OR define the area of acquisition, describing how the region was determined.*

Diffusion MRI ☐ Used ☐ Not used

Preprocessing

Preprocessing software	*Provide detail on software version and revision number and on specific parameters (model/functions, brain extraction, segmentation, smoothing kernel size, etc.).*
Normalization	*If data were normalized/standardized, describe the approach(es): specify linear or non-linear and define image types used for transformation OR indicate that data were not normalized and explain rationale for lack of normalization.*
Normalization template	*Describe the template used for normalization/transformation, specifying subject space or group standardized space (e.g. original Talairach, MNI305, ICBM152) OR indicate that the data were not normalized.*
Noise and artifact removal	*Describe your procedure(s) for artifact and structured noise removal, specifying motion parameters, tissue signals and physiological signals (heart rate, respiration).*
Volume censoring	*Define your software and/or method and criteria for volume censoring, and state the extent of such censoring.*

Statistical modeling & inference

Model type and settings	*Specify type (mass univariate, multivariate, RSA, predictive, etc.) and describe essential details of the model at the first and second levels (e.g. fixed, random or mixed effects; drift or auto-correlation).*
Effect(s) tested	*Define precise effect in terms of the task or stimulus conditions instead of psychological concepts and indicate whether ANOVA or factorial designs were used.*

Specify type of analysis: ☐ Whole brain ☐ ROI-based ☐ Both

Statistic type for inference (See Eklund et al. 2016)	*Specify voxel-wise or cluster-wise and report all relevant parameters for cluster-wise methods.*
Correction	*Describe the type of correction and how it is obtained for multiple comparisons (e.g. FWE, FDR, permutation or Monte Carlo).*

Models & analysis

n/a	Involved in the study
☐	☐ Functional and/or effective connectivity
☐	☐ Graph analysis
☐	☐ Multivariate modeling or predictive analysis

Functional and/or effective connectivity	*Report the measures of dependence used and the model details (e.g. Pearson correlation, partial correlation, mutual information).*
Graph analysis	*Report the dependent variable and connectivity measure, specifying weighted graph or binarized graph, subject- or group-level, and the global and/or node summaries used (e.g. clustering coefficient, efficiency, etc.).*
Multivariate modeling and predictive analysis	*Specify independent variables, features extraction and dimension reduction, model, training and evaluation metrics.*
